# Metaproteomic data of maize rhizosphere for deciphering functional diversity

**DOI:** 10.1016/j.dib.2019.104574

**Published:** 2019-09-27

**Authors:** Renu, Sanjay Kumar Gupta, Ashutosh Kumar Rai, Khan Mohd. Sarim, Anu Sharma, Neeraj Budhlakoti, Devendra Arora, Dhiraj Kumar Verma, Dhananjaya P. Singh

**Affiliations:** a ICAR-National Bureau of Agriculturally Important Microorganisms, Kushmaur, Maunath Bhanjan, 275 101, India; b Department of Biochemistry, College of Medicine, Imam Abdulrahman Bin Faisal University, Dammam, 31441, Saudi Arabia; c Centre for Agricultural Bioinformatics, ICAR-IASRI, New Delhi, 110012, India

**Keywords:** Metaproteome, Maize rhizosphere, Soil microbial proteins, Microbiota

## Abstract

Metaproteomics is a powerful tool for obtaining data on all proteins recovered directly from environmental samples at a given time. It provides a direct evidence of functional diversity and structure among microbiota present in niches and significant insights into microbial activity together with metabolomics, which is the study of the intermediate and end-products of cellular processes. Metaproteomics is a comparatively new approach which is facing a number of empirical, technical, computational and experimental design challenges that needs to be addressed. Presently only little efforts have been made to have information on microbial proteins in rhizospheric soil of maize through metagemonics approach but there is no direct evidence on functions of microbial community in this very important niche. Since rhizosphere microbiome plays important role in plant growth and development the present study is conducted to optimize the metaproteomic extraction protocol from maize rhizosphere and analyse functionality of microbial communities.

We present metaproteome data from maize rhizospheric soil. Isolation of metaproteome from maize rhizosphere collected from ICAR-IISS, Mau experimental Farm was done with the standardized protocol at our laboratory and metaproteome analysis was done with the standardized pipeline. In total 696 proteins with different functions representing 244 genus and 393 species were identified. The proteome data provides direct evidence on the biological processes in soil ecosystem and is the first reported reference data from maize rhizosphere. The LC MS/MS proteomic data are available *via* ProteomeXchange with identifier PXD014519.

**Table:** Specifications table

Subject	Biology
Specific subject area	Metaproteomics
Type of data	1) Mass spectrometry data (*.raw) 2) Search output data (*.mgf) 3) Excel file
How data were acquired	NanoAcquity UPLC BEH C18 for separation of peptides Waters Synapt G2 Q-TOF instrument for MS/MS
Data format	1) mgf (output files/peak files) 2) Search files (Excel files) 3) Raw data (.raw folder)
Parameters for data collection	Metaproteome isolation was done from rhizosphere of maize, cleavage of microbial proteins was done using trypsin and subsequently analyzed by LC-MS/MS
Description of data collection	1) Collection of maize rhizospheric soil 2) Metaproteome isolation 3) LC-MS/MS analysis
Data source location	Mau, Uttar Pradesh, India Sample collection: ICAR-Indian Institute of Seed Science Farm, Mau, India (26.128395^0^N 83.739273^0^E)
Data accessibility	Data is within this article. The mass spectrometry proteomics data have been deposited to the ProteomeXchange Consortium via the PRIDE [1] partner repository with the dataset identifier PXD014519. Project DOI: Not applicable

**Table:** 

**Value of the data** • First time reference set of proteomic data of microbial communities associated with maize rhizosphere. Identified expressed proteins matched by the species expressing them gives relevant information about system functioning, its microbiota and ecology of maize rhizosphere. • The data could be useful for researcher in understanding the physiology and adaptations of the plant associated microbiota and the actual interaction pathways occurring between roots and bacterial communities in the soil. • The data provides direct evidence on the biological processes in maize soil ecosystem and the repertoire of proteins secreted by microorganisms in rhizospheric zone which are used by them to compete and cooperate, and extends knowledge about the activity of the microbial community in association with plant root zone. The method could be extended to maize crop under different soils and/or stressed conditions and comparison with the reference data can provide clue on key protein players under different environmental conditions.

## 1 Data

**Fig. 1:**
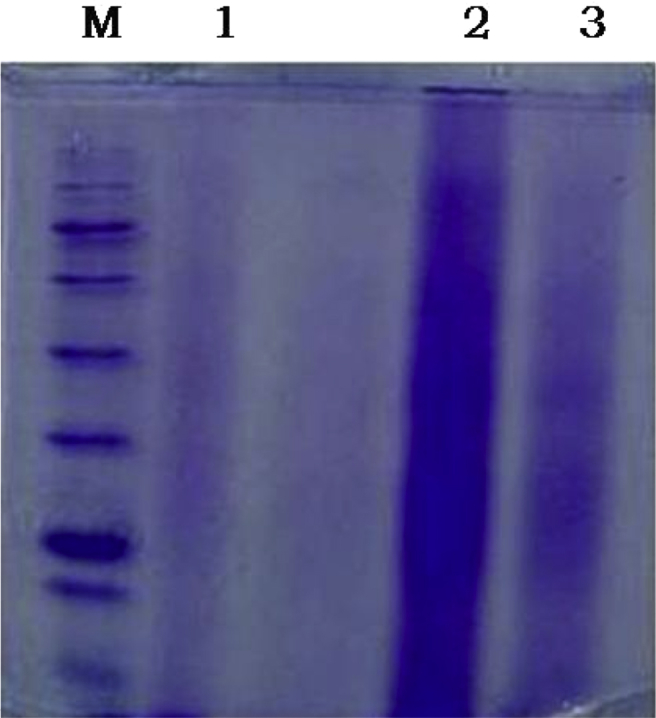
Protein samples from different extraction protocols on 12% SDS PAGE; lane M − 250 kDa Protein Ladder; 1-SDS Protocol (*Keiblinger* et al., 2012); 2-SDS-Phenol protocol with bead beating (our protocol); 3- NaOH Protocol (Benndorf*et al.*, 2007).

The dataset in this article describes the metaproteome which includes all proteins expressed by the microbial communities from rhizosphere of maize. Fig. 1 describes the protein yield obtained from our protocol and compares with earlier available protocols by loading equal volume of proteins by different protocols on 12% SDS PAGE.

### 1.1 LC-MS/MS analysis

**Fig. 2:**
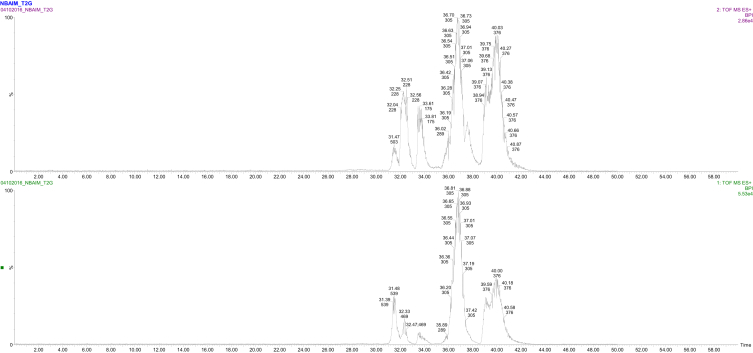
Chromatogram of metaproteome of maize rhizospheric soil.

**Fig. 3:**
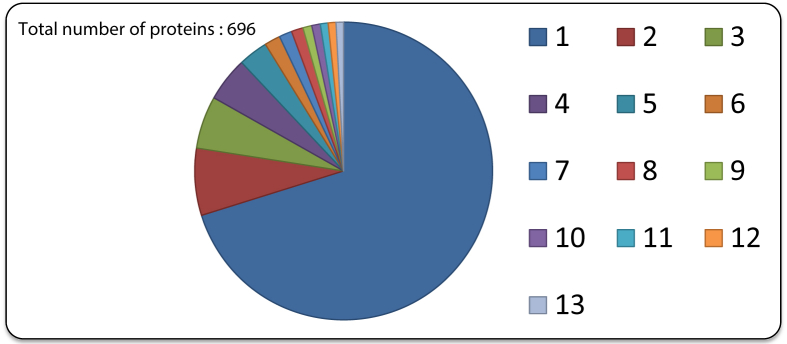
Relative abundance of functional proteins in metaproteome of maize rhizospheric soil sample (1) DNA-directed RNA polymerase, (2) Serine hydroxymethyltransferase, (3) Polyribonucleotide nucleotidyltransferase, (4) tRNA ligase, (5) ATP-dependent helicase, (6) UvrABC system protein, (7) Chromosome partition protein, (8) Phosphoribosylformylglycinamidine synthase, (9) DNA ligase, (10) Translation initiation factor, (11) Cytadherence high molecular weight protein, (12) Periplasmic nitrate reductase, (13) Nitrate reductase.

The chromatogram of metaproteome of maize rhizospheric soil obtained after LC-MS/MS is depicted by Fig. 2. The mass spectrometry proteomics data is provided on the ProteomeXchange Consortium *via* the PRIDE partner repository with the dataset identifier PXD014519 which contains mgf (output files/peak files); search files (Excel files) and raw data (.raw folder). Total numbers of 696 proteins representing 244 genus and 393 species were identified in dataset. Table S1 and S2 (supplementary excel file) describes the list of genus and species, respectively, representing total proteins in metaproteome. Fig. 3 and Table 1 describes the relative abundance of functional proteins obtained from metaproteome of maize rhizosphere. Protein functional analysis shows that DNA-directed RNA polymerase was predominating (64.47%). Table 2 contains list of number of proteins mapped on different metabolic pathways as in UniPathway. Eighty out of 696 identifiers from UniProtKB AC/ID were successfully mapped to 23 UniPathway IDs. Table S3 provides information on the biological functions of identified proteins. Dataset identifies the presence of membrane transporter protein including ABC transporter, bacterial secretion system and primarily two component systems which is a signal system allow bacteria to rapidly sense changes in environment stresses, altering growth condition. In addition serine hydroxymethyltransferase which plays a key role in metablolism of many key compound like glycine, serine and threonine; glyoxylate and dicarboxylate metabolism acid; methane; and biosynthesis of secondary metabolites, antibiotics and amino acids were abundant (6.69% relative abundance). Other key enzymes like catalase-peroxidase and nitrate and periplasmic nitrate reductase having role in stress response mechanism and plant growth promotion, respectively, were also present.

**Table 1:** Relative abundance of functional proteins in metaproteome of maize rhizospheric soil sample.

Functional Proteins	% Relative Abundance
DNA-directed RNA polymerase	64.47
Serine hydroxymethyltransferase	6.69
Polyribonucleotide nucleotidyltransferase	5.25
tRNA ligase	4.42
ATP-dependent helicase	2.95
UvrABC system protein	1.58
Chromosome partition protein	1.29
Phosphoribosylformylglycinamidine synthase	1.18
DNA ligase	0.88
Translation initiation factor	0.86
Cytadherence high molecular weight protein	0.78
Periplasmic nitrate reductase	0.78
Nitrate reductase	0.75
Other Proteins	8.14

**Table 2:** List of number of proteins mapped on different metabolic pathways as in UniPathway.

UniPathway ID	Pathway description	Number of proteins mapped
UPA00031	Histidine biosynthetic process	1
UPA00034	Biosynthesis of l-lysine through the diaminopimelate (DAP) pathway	1
UPA00048	Leucine biosynthetic process	1
UPA00050	l-threonine biosynthesis	1
UPA00051	l-methionine biosynthesis	1
UPA00060	Thiamine diphosphate biosynthetic process	1
UPA00074	de novo’ IMP biosynthetic process	13
UPA00078	Biotin biosynthesis	1
UPA00113	UDP-N-acetylglucosamine biosynthetic process	1
UPA00157	Beta-ketoadipate pathway	1
UPA00186	Agmatine biosynthesis	1
UPA00193	One-carbon metabolism; tetrahydrofolate interconversion	47
UPA00219	Peptidoglycan biosynthesis,	2
UPA00223	Tricarboxylic acid cycle	1
UPA00251	Protoporphyrin-IX biosynthesis∼Biosynthesis of protoporphyrin-IX, a porphyrin derivative precursor of heme and chlorphyll compounds	1
UPA00288	Glycine biosynthesis;	47
UPA00538	Protein modification; protein lipoylation via endogenous pathway	1
UPA00618	Regulation of glycerol uptake and metabolism	1
UPA00652	Denitrification pathway	1
UPA00671	Bacteriochlorophyll biosynthesis (light-independent)	2
UPA00863	Butanoate metabolism	1
UPA00973	Bacterial outer membrane biogenesis; LPS lipid A biosynthesis;	1
UPA01068	Cofactor metabolism	1

## 2 Experimental design, materials, and methods

### 2.1 Sampling

Soil samples from rhizosphere of maize (v*ar* Bio9637) at approximately 60 days of sowing at ICAR- Indian Institute of Seed Sciences Farm,Mau, India (26.128395^0^N 83.739273^0^E) was collected on August 9, 2015 representing the early reproductive (R) growth stages. Five plant samples were taken on average 1 m apart and pooled. The samples were transferred to the lab on dry ice, pre-processed and stored at −80 °C for downstream applications. Physico chemical analysis of soil was done (clay loam; pH 8.3; EC 0.83 dS/m; % Oxidizable organic carbon 1.18).

### 2.2 Metaroteome isolation and sample preparation

Optimization of metaproteome extraction protocol was done based on protocol by *Keiblinger* *et* *al*., 2012 [2] and Benndorf *et al.*, 2007 [3] with modifications at several steps. The soil samples were mixed with 10% (w/w) polyvinylpolypyrrolidone (PVPP) to clean samples from humic acids with a pestle in liquid nitrogen to minimize proteolysis and protein degradation. Then the soil samples were prewashed with 10% TCA/acetone to remove contaminants affecting the separation, 80% methanol washed to remove poly phenolic compounds and a final acetone washing was done. To remove the traces of acetone soil sample was incubated at 37 °C. To extract the protein, 2 g soil sample was taken and mixed with 1 g gerconium bead containing extraction buffer (1:1 (v/v) phenol (pH 8.0) and SDS phenol buffer (50 mM Tris, 1% SDS, pH 7.5)) by vortexing. The tubes were beat beaten at full speed for 30 secs. The supernatant was collected at 3220 g for 5 min at 4 °C. A second extraction step (sequential extraction) was done as described above. Extraction buffer was added in a 1:3 (w/v) ratio. The supernatant thus obtained from both the steps was pooled and centrifuged at 10640 g for 15 min at 4 °C. The lower phenolic phase containing protein was collected into a fresh tube. Phenolic phase was washed twice with chilled MQ water by vortexing for 5 min and centrifugation (at 10640 g for 20 min at 4 °C). The purified phenol phases were pooled and proteins were precipitated five time with ice chilled 0.1 M ammonium acetate in methanol treated overnight at −20 °C. Protein pellet was obtained by centrifugation at 10640*g* for 20 min at 4 °C. Supernatants were discarded. The pellets were first washed with 100% pre-chilled methanol and then by acetone by gentle vortexing as described above. The supernatant was discarded and the pellets were air dried. Pellets were resuspended in a maximum 250 μl of 50 mM ammonium acetate containing 0.1–0.5% SDS by pipetting up and down and left overnight at 4 °C.

The protein samples were centrifuged for 5 min at 17960 g, the resulting supernatant was used for further processing. Equal volumes of solubilized proteins were loaded on 12% SDS-PAGE gel electrophoresis to evaluate the protein separation pattern [4]. Our protocol showed better recovery of proteins than SDS and NaOH protocols [2, 3] (Fig. 1).

#### 2.2.1 Protein digestion

Protein sample (100 μg) was taken in 100 μl of 50 mM NH_4_HCO_3_ for trypsin digestion and pre-treated with 100 mM DTT at for 1hr at 95 °C followed by 250 mM iodoacetamide for 45 min at room temperature in dark. Finally, the sample was digested with trypsin (Promega) at final concentration of 2 μg/ml overnight at 37 °C.

The resulting sample was vacuum-dried at 37 °C. The pellet was dissolved in 10 μl of 0.1% formic acid in water. The whole content was centrifuged at 10000 g for 10 min at room temperature and supernatant was collected in a fresh tube.

#### 2.2.2 Peptides separation

The digested peptides were separated by reversed-phase nano-LC (Acquity Waters nUPLC system; column: BEH C-18, 75 μm × 150 mm X 1.7 μm; eluent: 0.1% formic acid, 0.1% acetonitrile) and analyzed by MS/MS. Chromatograms (Fig. 2) of the MS/MS spectra were analyzed. The raw data was processed by MassLynx 4.1 WATER software. Each spectrum was used to generate Mascot generic file (MGF).

#### 2.2.3 Bioinformatics analysis

MGF file generated against individual spectra was used to search in database for protein identification on MASCOT server. Search parameters were as follows: taxonomy, all entries; fixed modification, cysteine carbamidomethylation; variable modifications, methionine oxidation; enzyme, trypsin; maximum number of missed cleavages, 1; peptide tolerance, 50 ppm; and MS/MS tolerance, 100 ppm. The list of peptides, assigned by protein hit number, were catalogued into assigned proteins after microbiological filter and associated to OTUs.
