# Supplementary material for: Metaproteomic data of maize rhizosphere for deciphering functional diversity
Source: Data Brief. 2019 Sep 27;27:104574. doi: 10.1016/j.dib.2019.104574 (PMC6806416; doi:10.1016/j.dib.2019.104574)
Supplement: Multimedia component 3 [file mmc3.doc]

**Table S3: Dataset on functions and/or role of identified proteins present in the metaproteome of maize rhizosphere**

| **Uniprot Accession id** | **Protein name** | **Biological functions/pathways (source : KEGG)** |
| --- | --- | --- |
| Q13Z67 | 23S rRNA (uracil(1939)-C(5))-methyltransferase RlmD OS=Burkholderia xenovorans (strain LB400) GN=rlmD PE=3 SV=2 | Genetic information processing |
| A0JZ51 | 30S ribosomal protein S13 OS=Arthrobacter sp. (strain FB24) GN=rpsM PE=3 SV=1 | Ribosome subunit |
| A3CMJ2 | 3-isopropylmalate dehydratase large subunit OS=Streptococcus sanguinis (strain SK36) GN=leuC PE=3 SV=1 | Carbohydrate metabolism : C5-Branched dibasic acid metabolism; Amino acid metabolism: Valine, leucine and isoleucine biosynthesis |
| A4J5C3 | 5-methyltetrahydropteroyltriglutamate--homocysteine methyltransferase OS=Desulfotomaculum reducens (strain MI-1) GN=metE PE=3 SV=1 | drm00450 Selenocompound metabolism  drm01100 Metabolic pathways  drm01110Biosynthesis of secondary metabolites  drm01230 Biosynthesis of amino acids |
| A9WJN7 | 60 kDa chaperonin 1 OS=Chloroflexus aurantiacus (strain ATCC 29366 / DSM 635 / J-10-fl) GN=groL1 PE=3 SV=1 | cau03018 RNA degradation |
| Q2RL13 | 60 kDa chaperonin 1 OS=Moorella thermoacetica (strain ATCC 39073 / JCM 9320) GN=groL1 PE=3 SV=1 | mta03018 RNA degradation |
| A7NIK8 | 60 kDa chaperonin 1 OS=Roseiflexus castenholzii (strain DSM 13941 / HLO8) GN=groL1 PE=3 SV=1 | rca03018 RNA degradation |
| Q2RGL8 | 60 kDa chaperonin 2 OS=Moorella thermoacetica (strain ATCC 39073 / JCM 9320) GN=groL2 PE=3 SV=1 | mta03018 RNA degradation |
| A1R074 | 60 kDa chaperonin OS=Borrelia turicatae (strain 91E135) GN=groL PE=3 SV=1 | btu03018 RNA degradation |
| Q8R5T7 | 60 kDa chaperonin OS=Caldanaerobacter subterraneus subsp. tengcongensis (strain DSM 15242 / JCM 11007 / NBRC 100824 / MB4) GN=groL PE=3 SV=1 | tte03018 RNA degradation |
| B0TCA0 | 60 kDa chaperonin OS=Heliobacterium modesticaldum (strain ATCC 51547 / Ice1) GN=groL PE=3 SV=1 | hmo03018 RNA degradation |
| B4U8T6 | 60 kDa chaperonin OS=Hydrogenobaculum sp. (strain Y04AAS1) GN=groL PE=3 SV=1 | hya03018 RNA degradation |
| B2A5V3 | 60 kDa chaperonin OS=Natranaerobius thermophilus (strain ATCC BAA-1301 / DSM 18059 / JW/NM-WN-LF) GN=groL PE=3 SV=1 | nth03018 RNA degradation |
| Q8CQD9 | Accumulation-associated protein OS=Staphylococcus epidermidis (strain ATCC 12228) GN=SE_0175 PE=3 SV=1 | accumulation-associated protein  surface protein G |
| Q9WXC3 | Adenylate cyclase OS=Streptomyces griseus GN=cya PE=3 SV=1 | cAMP biosynthetic process, intracellular signal transduction |
| Q1D823 | Adventurous-gliding motility protein Z OS=Myxococcus xanthus (strain DK 1622) GN=aglZ PE=1 SV=1 | gliding motility protein AglZ |
| Q98Q12 | Alanine--tRNA ligase OS=Mycoplasma pulmonis (strain UAB CTIP) GN=alaS PE=3 SV=1 | mpu00970 Aminoacyl-tRNA biosynthesis |
| Q2G8V0 | Alanine--tRNA ligase OS=Novosphingobium aromaticivorans (strain ATCC 700278 / DSM 12444 / CIP 105152 / NBRC 16084 / F199) GN=alaS PE=3 SV=1 | nar00970 Aminoacyl-tRNA biosynthesis |
| A5V3L0 | Alanine--tRNA ligase OS=Sphingomonas wittichii (strain RW1 / DSM 6014 / JCM 10273) GN=alaS PE=3 SV=1 | swi00970 Aminoacyl-tRNA biosynthesis |
| Q1MFL8 | Aliphatic sulfonates import ATP-binding protein SsuB 1 OS=Rhizobium leguminosarum bv. viciae (strain 3841) GN=ssuB1 PE=3 SV=1 | rle00920 Sulfur metabolism  rle02010 ABC transporters |
| A2BTS0 | Aspartate--tRNA(Asp/Asn) ligase OS=Prochlorococcus marinus (strain AS9601) GN=aspS PE=3 SV=1 | pmb00970 Aminoacyl-tRNA biosynthesis |
| P94417 | Aspartokinase 3 OS=Bacillus subtilis (strain 168) GN=yclM PE=1 SV=1 | bsu00260 Glycine, serine and threonine metabolism  bsu00261 Monobactam biosynthesis  bsu00270 Cysteine and methionine metabolism  bsu00300 Lysine biosynthesis  bsu01100 Metabolic pathways  bsu01110 Biosynthesis of secondary metabolites  bsu01120 Microbial metabolism in diverse environments  bsu01130 Biosynthesis of antibiotics  bsu01210 2-Oxocarboxylic acid metabolism  bsu01230 Biosynthesis of amino acids |
| A7GM36 | ATP-dependent helicase/deoxyribonuclease subunit B OS=Bacillus cytotoxicus (strain DSM 22905 / CIP 110041 / 391-98 / NVH 391-98) GN=addB PE=3 SV=1 | DNA repair and recombination proteins |
| A9VJ01 | ATP-dependent helicase/deoxyribonuclease subunit B OS=Bacillus weihenstephanensis (strain KBAB4) GN=addB PE=3 SV=1 | DNA repair and recombination proteins |
| B8I2Y3 | ATP-dependent helicase/deoxyribonuclease subunit B OS=Clostridium cellulolyticum (strain ATCC 35319 / DSM 5812 / JCM 6584 / H10) GN=addB PE=3 SV=1 | DNA repair and recombination proteins |
| Q8XPE3 | ATP-dependent helicase/deoxyribonuclease subunit B OS=Clostridium perfringens (strain 13 / Type A) GN=addB PE=3 SV=1 | DNA repair and recombination proteins |
| Q0SWW7 | ATP-dependent helicase/deoxyribonuclease subunit B OS=Clostridium perfringens (strain SM101 / Type A) GN=addB PE=3 SV=1 | DNA repair and recombination proteins |
| B1KUZ8 | ATP-dependent helicase/nuclease subunit A OS=Clostridium botulinum (strain Loch Maree / Type A3) GN=addA PE=3 SV=1 | DNA repair and recombination proteins |
| B1I493 | ATP-dependent helicase/nuclease subunit A OS=Desulforudis audaxviator (strain MP104C) GN=addA PE=3 SV=1 | DNA repair and recombination proteins |
| A8AY33 | ATP-dependent helicase/nuclease subunit A OS=Streptococcus gordonii (strain Challis / ATCC 35105 / BCRC 15272 / CH1 / DL1 / V288) GN=addA PE=3 SV=1 | DNA repair and recombination proteins |
| Q8VPF1 | Beta-ketoadipyl-CoA thiolase OS=Pseudomonas knackmussii (strain DSM 6978 / LMG 23759 / B13) GN=pcaF PE=1 SV=1 | beta-ketoadipate pathway |
| Q7VT27 | Bifunctional protein GlmU OS=Bordetella pertussis (strain Tohama I / ATCC BAA-589 / NCTC 13251) GN=glmU PE=3 SV=1 | bpe00520 Amino sugar and nucleotide sugar metabolism  bpe01100 Metabolic pathways  bpe01130 Biosynthesis of antibiotics |
| Q7TTX6 | Bifunctional purine biosynthesis protein PurH OS=Synechococcus sp. (strain WH8102) GN=purH PE=3 SV=1 | syw00230 Purine metabolism  syw00670 One carbon pool by folate  syw01100 Metabolic pathways  syw01110 Biosynthesis of secondary metabolites  syw01130 Biosynthesis of antibiotics |
| C4K6F4 | Biosynthetic arginine decarboxylase OS=Hamiltonella defensa subsp. Acyrthosiphon pisum (strain 5AT) GN=speA PE=3 SV=1 | hde00330 Arginine and proline metabolism  hde01100 Metabolic pathways |
| B2GLQ7 | Biotin synthase OS=Kocuria rhizophila (strain ATCC 9341 / DSM 348 / NBRC 103217 / DC2201) GN=bioB PE=3 SV=1 | krh00780 Biotin metabolism  krh01100 Metabolic pathways |
| Q6GJA6 | Bone sialoprotein-binding protein OS=Staphylococcus aureus (strain MRSA252) GN=bbp PE=3 SV=1 | sar05150  Staphylococcus aureus infection |
| A4SS04 | Catalase-peroxidase OS=Aeromonas salmonicida (strain A449) GN=katG PE=3 SV=1 | asa00360 Phenylalanine metabolism  asa00380 Tryptophan metabolism  asa01100 Metabolic pathways  asa01110 Biosynthesis of secondary metabolites |
| Q4G4B1 | Catalase-peroxidase OS=Edwardsiella tarda GN=katG PE=3 SV=1 | Response to oxidative stress, Hydrogen peroxide catabolic process |
| B1MBD0 | Catalase-peroxidase OS=Mycobacterium abscessus (strain ATCC 19977 / DSM 44196 / CIP 104536 / JCM 13569 / NCTC 13031 / TMC 1543) GN=katG PE=3 SV=1 | response to oxidative stress, hydrogen peroxide catabolic process |
| Q73ZD5 | Catalase-peroxidase OS=Mycobacterium paratuberculosis (strain ATCC BAA-968 / K-10) GN=katG PE=3 SV=1 | mpa00360 Phenylalanine metabolism  mpa00380 Tryptophan metabolism  mpa00983 Drug metabolism otherenzymes  mpa01100 Metabolic pathways  mpa01110 Biosynthesis of secondary metabolites |
| B1XK45 | Catalase-peroxidase OS=Synechococcus sp. (strain ATCC 27264 / PCC 7002 / PR-6) GN=katG PE=3 SV=1 | syp00360 Phenylalanine metabolism  syp00380 Tryptophan metabolism  syp01100 Metabolic pathways  syp01110 Biosynthesis of secondary metabolites |
| A5F7X7 | Catalase-peroxidase OS=Vibrio cholerae serotype O1 (strain ATCC 39541 / Classical Ogawa 395 / O395) GN=katG PE=3 SV=1 | vco00360 Phenylalanine metabolism  vco00380 Tryptophan metabolism  vco01100 Metabolic pathways  vco01110 Biosynthesis of secondary metabolites |
| P54423 | Cell wall-associated protease OS=Bacillus subtilis (strain 168) GN=wprA PE=1 SV=2 | genetic information processing |
| Q7VYV6 | Chaperone protein ClpB OS=Bordetella pertussis (strain Tohama I / ATCC BAA-589 / NCTC 13251) GN=clpB PE=3 SV=1 | HSP Stress resistence gene |
| Q5P1H7 | Chaperone protein DnaJ 2 OS=Aromatoleum aromaticum (strain EbN1) GN=dnaJ2 PE=3 SV=1 | genetic information processing |
| P60068 | Chlorate reductase subunit alpha OS=Ideonella dechloratans GN=clrA PE=1 SV=1 | respiratory oxidant |
| Q5PB48 | Chromosomal replication initiator protein DnaA OS=Anaplasma marginale (strain St. Maries) GN=dnaA PE=3 SV=1 | Two component system |
| A7MEV9 | Chromosome partition protein MukB OS=Cronobacter sakazakii (strain ATCC BAA-894) GN=mukB PE=3 SV=1 | genetic information processing |
| C5BAC7 | Chromosome partition protein MukB OS=Edwardsiella ictaluri (strain 93-146) GN=mukB PE=3 SV=1 | genetic information processing |
| B2VC99 | Chromosome partition protein MukB OS=Erwinia tasmaniensis (strain DSM 17950 / CIP 109463 / Et1/99) GN=mukB PE=3 SV=1 | genetic information processing |
| A1A9K1 | Chromosome partition protein MukB OS=Escherichia coli O1:K1 / APEC GN=mukB PE=3 SV=1 | genetic information processing |
| C6DFB1 | Chromosome partition protein MukB OS=Pectobacterium carotovorum subsp. carotovorum (strain PC1) GN=mukB PE=3 SV=1 | genetic information processing |
| Q2NU97 | Chromosome partition protein MukB OS=Sodalis glossinidius (strain morsitans) GN=mukB PE=3 SV=1 | genetic information processing |
| A4TN03 | Chromosome partition protein MukB OS=Yersinia pestis (strain Pestoides F) GN=mukB PE=3 SV=1 | genetic information processing |
| Q8KBS6 | Chromosome partition protein Smc OS=Chlorobium tepidum (strain ATCC 49652 / DSM 12025 / NBRC 103806 / TLS) GN=smc PE=3 SV=1 | genetic information processing |
| C4ZJU1 | Chromosome partition protein Smc OS=Thauera sp. (strain MZ1T) GN=smc PE=3 SV=2 | genetic information processing |
| Q44349 | Conjugal transfer protein TraA OS=Agrobacterium fabrum (strain C58 / ATCC 33970) GN=traA PE=3 SV=2 | Non sexual transfer of genetic materia |
| A9IRQ9 | Cysteine--tRNA ligase OS=Bartonella tribocorum (strain CIP 105476 / IBS 506) GN=cysS PE=3 SV=1 | Cysteine biosynthesis |
| P47460 | Cytadherence high molecular weight protein 2 OS=Mycoplasma genitalium (strain ATCC 33530 / G-37 / NCTC 10195) GN=hmw2 PE=3 SV=1 | promots export of HMW1 to the cell surface |
| P75471 | Cytadherence high molecular weight protein 2 OS=Mycoplasma pneumoniae (strain ATCC 29342 / M129) GN=hmw2 PE=1 SV=1 | Cytadherence protein |
| Q1LLY7 | D-(-)-3-hydroxybutyrate oligomer hydrolase OS=Cupriavidus metallidurans (strain ATCC 43123 / DSM 2839 / NBRC 102507 / CH34) GN=Rmet_1960 PE=3 SV=1 | rme00650 Butanoate metabolism  rme01100 Metabolic pathways |
| B7L771 | DNA ligase B OS=Escherichia coli (strain 55989 / EAEC) GN=ligB PE=3 SV=1 | eck03030 DNA replication  eck03410 Base excision repair  eck03420 Nucleotide excision repair  eck03430 Mismatch repair |
| B7MFK8 | DNA ligase B OS=Escherichia coli O45:K1 (strain S88 / ExPEC) GN=ligB PE=3 SV=1 | ecz03030 DNA replication  ecz03410 Base excision repair  ecz03420 Nucleotide excision repair  ecz03430 Mismatch repair |
| Q0SYH4 | DNA ligase B OS=Shigella flexneri serotype 5b (strain 8401) GN=ligB PE=3 SV=2 | eck03030 DNA replication  eck03410 Base excision repair  eck03420 Nucleotide excision repair  eck03430 Mismatch repair |
| B2UL69 | DNA ligase OS=Akkermansia muciniphila (strain ATCC BAA-835 / Muc) GN=ligA PE=3 SV=1 | eck03030 DNA replication  eck03410 Base excision repair  eck03420 Nucleotide excision repair  eck03430 Mismatch repair |
| Q0VR01 | DNA ligase OS=Alcanivorax borkumensis (strain ATCC 700651 / DSM 11573 / NCIMB 13689 / SK2) GN=ligA PE=3 SV=1 | eck03030 DNA replication  eck03410 Base excision repair  eck03420 Nucleotide excision repair  eck03430 Mismatch repair |
| Q470D2 | DNA ligase OS=Cupriavidus pinatubonensis (strain JMP 134 / LMG 1197) GN=ligA PE=3 SV=1 | eck03030 DNA replication  eck03410 Base excision repair  eck03420 Nucleotide excision repair  eck03430 Mismatch repair |
| B2GL64 | DNA ligase OS=Kocuria rhizophila (strain ATCC 9341 / DSM 348 / NBRC 103217 / DC2201) GN=ligA PE=3 SV=1 | eck03030 DNA replication  eck03410 Base excision repair  eck03420 Nucleotide excision repair  eck03430 Mismatch repair |
| A5VLG6 | DNA ligase OS=Lactobacillus reuteri (strain DSM 20016) GN=ligA PE=3 SV=1 | eck03030 DNA replication  eck03410 Base excision repair  eck03420 Nucleotide excision repair  eck03430 Mismatch repair |
| Q82TW6 | DNA ligase OS=Nitrosomonas europaea (strain ATCC 19718 / CIP 103999 / KCTC 2705 / NBRC 14298) GN=ligA PE=3 SV=1 | eck03030 DNA replication  eck03410 Base excision repair  eck03420 Nucleotide excision repair  eck03430 Mismatch repair |
| C0ZXK3 | DNA ligase OS=Rhodococcus erythropolis (strain PR4 / NBRC 100887) GN=ligA PE=3 SV=1 | eck03030 DNA replication  eck03410 Base excision repair  eck03420 Nucleotide excision repair  eck03430 Mismatch repair |
| C1B1S4 | DNA ligase OS=Rhodococcus opacus (strain B4) GN=ligA PE=3 SV=1 | eck03030 DNA replication  eck03410 Base excision repair  eck03420 Nucleotide excision repair  eck03430 Mismatch repair |
| Q88UZ7 | DNA mismatch repair protein MutS OS=Lactobacillus plantarum (strain ATCC BAA-793 / NCIMB 8826 / WCFS1) GN=mutS PE=3 SV=1 | lpl03430 Mismatch repair |
| Q1D5J6 | DNA mismatch repair protein MutS OS=Myxococcus xanthus (strain DK 1622) GN=mutS PE=3 SV=1 | lpl03430 Mismatch repair |
| Q03FS8 | DNA polymerase III PolC-type OS=Pediococcus pentosaceus (strain ATCC 25745 / CCUG 21536 / LMG 10740 / 183-1w) GN=polC PE=3 SV=1 | ppe03030 DNA replication  ppe03430 Mismatch repair  ppe03440 Homologous recombination |
| Q5HPS7 | DNA polymerase III PolC-type OS=Staphylococcus epidermidis (strain ATCC 35984 / RP62A) GN=polC PE=3 SV=2 | ppe03030 DNA replication  ppe03430 Mismatch repair  ppe03440 Homologous recombination |
| Q4L5W6 | DNA polymerase III PolC-type OS=Staphylococcus haemolyticus (strain JCSC1435) GN=polC PE=3 SV=1 | ppe03030 DNA replication  ppe03430 Mismatch repair  ppe03440 Homologous recombination |
| Q9ZHF6 | DNA polymerase III PolC-type OS=Thermotoga maritima (strain ATCC 43589 / MSB8 / DSM 3109 / JCM 10099) GN=polC PE=1 SV=1 | ppe03030 DNA replication  ppe03430 Mismatch repair  ppe03440 Homologous recombination |
| A5IJJ8 | DNA polymerase III PolC-type OS=Thermotoga petrophila (strain RKU-1 / ATCC BAA-488 / DSM 13995) GN=polC PE=3 SV=1 | ppe03030 DNA replication  ppe03430 Mismatch repair  ppe03440 Homologous recombination |
| Q9XDH5 | DNA polymerase III subunit alpha OS=Thermus aquaticus GN=dnaE PE=1 SV=1 | ppe03030 DNA replication  ppe03430 Mismatch repair  ppe03440 Homologous recombination |
| P77917 | DNA-directed RNA polymerase subunit beta' (Fragment) OS=Pediococcus acidilactici GN=rpoC PE=3 SV=2 | RNA polymerase |
| Q5YQP4 | DNA-directed RNA polymerase subunit beta 1 OS=Nocardia farcinica (strain IFM 10152) GN=rpoB1 PE=3 SV=1 | nfa03020 RNA polymerase |
| A5FZX1 | DNA-directed RNA polymerase subunit beta OS=Acidiphilium cryptum (strain JF-5) GN=rpoB PE=3 SV=1 | acr03020 RNA polymerase |
| A5FZX0 | DNA-directed RNA polymerase subunit beta' OS=Acidiphilium cryptum (strain JF-5) GN=rpoC PE=3 SV=1 | acr03020 RNA polymerase |
| B7J460 | DNA-directed RNA polymerase subunit beta OS=Acidithiobacillus ferrooxidans (strain ATCC 23270 / DSM 14882 / CIP 104768 / NCIMB 8455) GN=rpoB PE=3 SV=1 | afr03020 RNA polymerase |
| A1TVT0 | DNA-directed RNA polymerase subunit beta OS=Acidovorax citrulli (strain AAC00-1) GN=rpoB PE=3 SV=1 | aav03020 RNA polymerase |
| B9MH46 | DNA-directed RNA polymerase subunit beta' OS=Acidovorax ebreus (strain TPSY) GN=rpoC PE=3 SV=1 | dia03020 RNA polymerase |
| A3M1G3 | DNA-directed RNA polymerase subunit beta OS=Acinetobacter baumannii (strain ATCC 17978 / CIP 53.77 / LMG 1025 / NCDC KC755 / 5377) GN=rpoB PE=3 SV=2 | acb03020 RNA polymerase |
| B0VDH9 | DNA-directed RNA polymerase subunit beta' OS=Acinetobacter baumannii (strain AYE) GN=rpoC PE=3 SV=1 | aby03020 RNA polymerase |
| A4SHV0 | DNA-directed RNA polymerase subunit beta' OS=Aeromonas salmonicida (strain A449) GN=rpoC PE=3 SV=1 | asa03020 RNA polymerase |
| Q8UE08 | DNA-directed RNA polymerase subunit beta OS=Agrobacterium fabrum (strain C58 / ATCC 33970) GN=rpoB PE=3 SV=2 | atu03020 RNA polymerase |
| Q8UE09 | DNA-directed RNA polymerase subunit beta' OS=Agrobacterium fabrum (strain C58 / ATCC 33970) GN=rpoC PE=3 SV=2 | atu03020 RNA polymerase |
| B2UQY1 | DNA-directed RNA polymerase subunit beta' OS=Akkermansia muciniphila (strain ATCC BAA-835 / Muc) GN=rpoC PE=3 SV=1 | amu03020 RNA polymerase |
| Q0VSM2 | DNA-directed RNA polymerase subunit beta OS=Alcanivorax borkumensis (strain ATCC 700651 / DSM 11573 / NCIMB 13689 / SK2) GN=rpoB PE=3 SV=1 | abo03020 RNA polymerase |
| Q0ABI2 | DNA-directed RNA polymerase subunit beta OS=Alkalilimnicola ehrlichii (strain ATCC BAA-1101 / DSM 17681 / MLHE-1) GN=rpoB PE=3 SV=1 | aeh03020 RNA polymerase |
| Q0ABI1 | DNA-directed RNA polymerase subunit beta' OS=Alkalilimnicola ehrlichii (strain ATCC BAA-1101 / DSM 17681 / MLHE-1) GN=rpoC PE=3 SV=1 | aeh03020 RNA polymerase |
| A6TWJ0 | DNA-directed RNA polymerase subunit beta OS=Alkaliphilus metalliredigens (strain QYMF) GN=rpoB PE=3 SV=1 | amt03020 RNA polymerase |
| B3ETY9 | DNA-directed RNA polymerase subunit beta' OS=Amoebophilus asiaticus (strain 5a2) GN=rpoC PE=3 SV=1 | aas03020 RNA polymerase |
| Q3M5D0 | DNA-directed RNA polymerase subunit beta OS=Anabaena variabilis (strain ATCC 29413 / PCC 7937) GN=rpoB PE=3 SV=1 | ava03020 RNA polymerase |
| Q2II86 | DNA-directed RNA polymerase subunit beta OS=Anaeromyxobacter dehalogenans (strain 2CP-C) GN=rpoB PE=3 SV=1 | ade03020 RNA polymerase |
| Q2II85 | DNA-directed RNA polymerase subunit beta' OS=Anaeromyxobacter dehalogenans (strain 2CP-C) GN=rpoC PE=3 SV=1 | ade03020 RNA polymerase |
| O67762 | DNA-directed RNA polymerase subunit beta OS=Aquifex aeolicus (strain VF5) GN=rpoB PE=3 SV=1 | aae03020 RNA polymerase |
| O67763 | DNA-directed RNA polymerase subunit beta' OS=Aquifex aeolicus (strain VF5) GN=rpoC PE=3 SV=1 | aae03020 RNA polymerase |
| A8EVZ3 | DNA-directed RNA polymerase subunit beta' OS=Arcobacter butzleri (strain RM4018) GN=rpoC PE=3 SV=1 | abu03020 RNA polymerase |
| Q5P339 | DNA-directed RNA polymerase subunit beta OS=Aromatoleum aromaticum (strain EbN1) GN=rpoB PE=3 SV=1 | eba03020 RNA polymerase |
| Q5P338 | DNA-directed RNA polymerase subunit beta' OS=Aromatoleum aromaticum (strain EbN1) GN=rpoC PE=3 SV=1 | eba03020 RNA polymerase |
| A1R8V3 | DNA-directed RNA polymerase subunit beta' OS=Arthrobacter aurescens (strain TC1) GN=rpoC PE=3 SV=1 | aau03020 RNA polymerase |
| A1KB34 | DNA-directed RNA polymerase subunit beta OS=Azoarcus sp. (strain BH72) GN=rpoB PE=3 SV=1 | azo03020 RNA polymerase |
| A1KB33 | DNA-directed RNA polymerase subunit beta' OS=Azoarcus sp. (strain BH72) GN=rpoC PE=3 SV=1 | azo03020 RNA polymerase |
| B6YPZ6 | DNA-directed RNA polymerase subunit beta' OS=Azobacteroides pseudotrichonymphae genomovar. CFP2 GN=rpoC PE=3 SV=1 | aps03020 RNA polymerase |
| A8HTY8 | DNA-directed RNA polymerase subunit beta OS=Azorhizobium caulinodans (strain ATCC 43989 / DSM 5975 / JCM 20966 / NBRC 14845 / NCIMB 13405 / ORS 571) GN=rpoB PE=3 SV=1 | azo03020 RNA polymerase |
| A8HTZ1 | DNA-directed RNA polymerase subunit beta' OS=Azorhizobium caulinodans (strain ATCC 43989 / DSM 5975 / JCM 20966 / NBRC 14845 / NCIMB 13405 / ORS 571) GN=rpoC PE=3 SV=1 | azo03020 RNA polymerase |
| Q5WLS0 | DNA-directed RNA polymerase subunit beta OS=Bacillus clausii (strain KSM-K16) GN=rpoB PE=3 SV=1 | bcl03020 RNA polymerase |
| Q5WLR9 | DNA-directed RNA polymerase subunit beta' OS=Bacillus clausii (strain KSM-K16) GN=rpoC PE=3 SV=1 | bcl03020 RNA polymerase |
| Q9Z9M2 | DNA-directed RNA polymerase subunit beta OS=Bacillus halodurans (strain ATCC BAA-125 / DSM 18197 / FERM 7344 / JCM 9153 / C-125) GN=rpoB PE=3 SV=1 | bha03020 RNA polymerase |
| Q9Z9M1 | DNA-directed RNA polymerase subunit beta' OS=Bacillus halodurans (strain ATCC BAA-125 / DSM 18197 / FERM 7344 / JCM 9153 / C-125) GN=rpoC PE=3 SV=1 | bha03020 RNA polymerase |
| A8F976 | DNA-directed RNA polymerase subunit beta OS=Bacillus pumilus (strain SAFR-032) GN=rpoB PE=3 SV=1 | bpu03020 RNA polymerase |
| P37870 | DNA-directed RNA polymerase subunit beta OS=Bacillus subtilis (strain 168) GN=rpoB PE=1 SV=2 | bsu03020 RNA polymerase |
| A0R8H3 | DNA-directed RNA polymerase subunit beta' OS=Bacillus thuringiensis (strain Al Hakam) GN=rpoC PE=3 SV=1 | btl03020 RNA polymerase |
| Q5L897 | DNA-directed RNA polymerase subunit beta OS=Bacteroides fragilis (strain ATCC 25285/ DSM 2151 / JCM 11019 / NCTC 9343) GN=rpoB PE=3 SV=1 | bfs03020 RNA polymerase |
| Q5L898 | DNA-directed RNA polymerase subunit beta' OS=Bacteroides fragilis (strain ATCC 25285 / DSM 2151 / JCM 11019 / NCTC 9343) GN=rpoC PE=3 SV=1 | bfs03020 RNA polymerase |
| Q8A470 | DNA-directed RNA polymerase subunit beta' OS=Bacteroides thetaiotaomicron (strain ATCC 29148 / DSM 2079 / NCTC 10582 / E50 / VPI-5482) GN=rpoC PE=3 SV=1 | th03020 RNA polymerase |
| A1USC9 | DNA-directed RNA polymerase subunit beta' OS=Bartonella bacilliformis (strain ATCC 35685 / KC583) GN=rpoC PE=3 SV=1 | RNA polymerase subunit |
| A9ISG4 | DNA-directed RNA polymerase subunit beta' OS=Bartonella tribocorum (strain CIP 105476 / IBS 506) GN=rpoC PE=3 SV=1 | btr03020 RNA polymerase |
| Q1LSX7 | DNA-directed RNA polymerase subunit beta OS=Baumannia cicadellinicola subsp. Homalodisca coagulata GN=rpoB PE=3 SV=1 | bci03020 RNA polymerase |
| Q1LSX6 | DNA-directed RNA polymerase subunit beta' OS=Baumannia cicadellinicola subsp. Homalodisca coagulata GN=rpoC PE=3 SV=1 | bci03020 RNA polymerase |
| Q6MJ10 | DNA-directed RNA polymerase subunit beta' OS=Bdellovibrio bacteriovorus (strain ATCC 15356 / DSM 50701 / NCIB 9529 / HD100) GN=rpoC PE=3 SV=1 | bba03020 RNA polymerase |
| B2IK56 | DNA-directed RNA polymerase subunit beta' OS=Beijerinckia indica subsp. indica (strain ATCC 9039 / DSM 1715 / NCIB 8712) GN=rpoC PE=3 SV=1 | bid03020 RNA polymerase |
| A1A317 | DNA-directed RNA polymerase subunit beta OS=Bifidobacterium adolescentis (strain ATCC 15703 / DSM 20083 / NCTC 11814 / E194a) GN=rpoB PE=3 SV=2 | bad03020 RNA polymerase |
| Q7VRP7 | DNA-directed RNA polymerase subunit beta OS=Blochmannia floridanus GN=rpoB PE=3 SV=1 | bfl03020 RNA polymerase |
| Q492B9 | DNA-directed RNA polymerase subunit beta OS=Blochmannia pennsylvanicus (strain BPEN) GN=rpoB PE=3 SV=1 | bpn03020 RNA polymerase |
| Q2L2M3 | DNA-directed RNA polymerase subunit beta OS=Bordetella avium (strain 197N) GN=rpoB PE=3 SV=2 | bav03020 RNA polymerase |
| Q7W0R9 | DNA-directed RNA polymerase subunit beta OS=Bordetella pertussis (strain Tohama I / ATCC BAA-589 / NCTC 13251) GN=rpoB PE=3 SV=1 | bpe03020 RNA polymerase |
| A9IJ22 | DNA-directed RNA polymerase subunit beta' OS=Bordetella petrii (strain ATCC BAA-461 / DSM 12804 / CCUG 43448) GN=rpoC PE=3 SV=1 | bpt03020 RNA polymerase |
| B7J1W0 | DNA-directed RNA polymerase subunit beta' OS=Borrelia burgdorferi (strain ZS7) GN=rpoC PE=3 SV=1 | bbz03020 RNA polymerase |
| B2S091 | DNA-directed RNA polymerase subunit beta' OS=Borrelia hermsii (strain HS1 / DAH) GN=rpoC PE=3 SV=1 | bhr03020 RNA polymerase |
| A1QZH7 | DNA-directed RNA polymerase subunit beta OS=Borrelia turicatae (strain 91E135) GN=rpoB PE=3 SV=1 | btu03020 RNA polymerase |
| A4YSI2 | DNA-directed RNA polymerase subunit beta' OS=Bradyrhizobium sp. (strain ORS 278) GN=rpoC PE=3 SV=1 | bra03020 RNA polymerase |
| C0ZIH0 | DNA-directed RNA polymerase subunit beta OS=Brevibacillus brevis (strain 47 / JCM 6285 / NBRC 100599) GN=rpoB PE=3 SV=1 | bbe03020 RNA polymerase |
| Q8YHP7 | DNA-directed RNA polymerase subunit beta' OS=Brucella melitensis biotype 1 (strain 16M / ATCC 23456 / NCTC 10094) GN=rpoC PE=3 SV=1 | bme03020 RNA polymerase |
| Q89B21 | DNA-directed RNA polymerase subunit beta' OS=Buchnera aphidicola subsp. Baizongia pistaciae (strain Bp) GN=rpoC PE=3 SV=1 | bab03020 RNA polymerase |
| Q058E6 | DNA-directed RNA polymerase subunit beta' OS=Buchnera aphidicola subsp. Cinara cedri (strain Cc) GN=rpoC PE=3 SV=1 | bcc03020 RNA polymerase |
| B1JU14 | DNA-directed RNA polymerase subunit beta OS=Burkholderia cenocepacia (strain MC0-3) GN=rpoB PE=3 SV=1 | bcm03020 RNA polymerase |
| Q13TG3 | DNA-directed RNA polymerase subunit beta' OS=Burkholderia xenovorans (strain LB400) GN=rpoC PE=3 SV=1 | bxb03020 RNA polymerase |
| A0RQI4 | DNA-directed RNA polymerase subunit beta' OS=Campylobacter fetus subsp. fetus (strain 82-40) GN=rpoC PE=3 SV=1 | cff03020 RNA polymerase |
| A1VYJ4 | DNA-directed RNA polymerase subunit beta OS=Campylobacter jejuni subsp. jejuni serotype O:23/36 (strain 81-176) GN=rpoB PE=3 SV=1 | cjj03020 RNA polymerase |
| A8FKR4 | DNA-directed RNA polymerase subunit beta' OS=Campylobacter jejuni subsp. jejuni serotype O:6 (strain 81116 / NCTC 11828) GN=rpoC PE=3 SV=1 | cju03020 RNA polymerase |
| Q3A9Q7 | DNA-directed RNA polymerase subunit beta OS=Carboxydothermus hydrogenoformans (strain ATCC BAA-161 / DSM 6008 / Z-2901) GN=rpoB PE=3 SV=1 | chy03020 RNA polymerase |
| Q05FH9 | DNA-directed RNA polymerase subunit beta' OS=Carsonella ruddii (strain PV) GN=rpoC PE=3 SV=1 | crp03020 RNA polymerase |
| B0SUP8 | DNA-directed RNA polymerase subunit beta' OS=Caulobacter sp. (strain K31) GN=rpoC PE=3 SV=1 | cak03020 RNA polymerase |
| B3PK30 | DNA-directed RNA polymerase subunit beta OS=Cellvibrio japonicus (strain Ueda107) GN=rpoB PE=3 SV=1 | cja03020 RNA polymerase |
| Q5L5I3 | DNA-directed RNA polymerase subunit beta OS=Chlamydia abortus (strain DSM 27085 / S26/3) GN=rpoB PE=3 SV=1 | cab03020 RNA polymerase |
| Q255E5 | DNA-directed RNA polymerase subunit beta' OS=Chlamydia felis (strain Fe/C-56) GN=rpoC PE=3 SV=1 | cfe03020 RNA polymerase |
| B3QQS1 | DNA-directed RNA polymerase subunit beta' OS=Chlorobaculum parvum (strain NCIB 8327) GN=rpoC PE=3 SV=1 | cpc03020 RNA polymerase |
| B3EER3 | DNA-directed RNA polymerase subunit beta' OS=Chlorobium limicola (strain DSM 245 / NBRC 103803 / 6330) GN=rpoC PE=3 SV=1 | cli03020 RNA polymerase |
| B3EL62 | DNA-directed RNA polymerase subunit beta' OS=Chlorobium phaeobacteroides (strain BS1) GN=rpoC PE=3 SV=1 | cpb03020 RNA polymerase |
| A1BD23 | DNA-directed RNA polymerase subunit beta OS=Chlorobium phaeobacteroides (strain DSM 266) GN=rpoB PE=3 SV=1 | cph03020 RNA polymerase |
| Q8KG14 | DNA-directed RNA polymerase subunit beta' OS=Chlorobium tepidum (strain ATCC 49652 / DSM 12025 / NBRC 103806 / TLS) GN=rpoC PE=3 SV=1 | cte03020 RNA polymerase |
| B8G4U8 | DNA-directed RNA polymerase subunit beta' OS=Chloroflexus aggregans (strain MD-66 / DSM 9485) GN=rpoC PE=3 SV=1 | cag03020 RNA polymerase |
| B9LL91 | DNA-directed RNA polymerase subunit beta OS=Chloroflexus aurantiacus (strain ATCC 29364 / DSM 637 / Y-400-fl) GN=rpoB PE=3 SV=1 | chl03020 RNA polymerase |
| A9WH11 | DNA-directed RNA polymerase subunit beta' OS=Chloroflexus aurantiacus (strain ATCC 29366 / DSM 635 / J-10-fl) GN=rpoC PE=3 SV=1 | cau03020 RNA polymerase |
| B3QYL5 | DNA-directed RNA polymerase subunit beta' OS=Chloroherpeton thalassium (strain ATCC 35110 / GB-78) GN=rpoC PE=3 SV=1 | cts03020 RNA polymerase |
| Q7NQE6 | DNA-directed RNA polymerase subunit beta OS=Chromobacterium violaceum (strain ATCC 12472 / DSM 30191 / JCM 1249 / NBRC 12614 / NCIMB 9131 / NCTC 9757) GN=rpoB PE=3 SV=1 | cvi03020 RNA polymerase |
| Q7NQE7 | DNA-directed RNA polymerase subunit beta' OS=Chromobacterium violaceum (strain ATCC 12472 / DSM 30191 / JCM 1249 / NBRC 12614 / NCIMB 9131 / NCTC 9757) GN=rpoC PE=3 SV=1 | cvi03020 RNA polymerase |
| Q1R0I2 | DNA-directed RNA polymerase subunit beta OS=Chromohalobacter salexigens (strain DSM 3043 / ATCC BAA-138 / NCIMB 13768) GN=rpoB PE=3 SV=1 | csa03020 RNA polymerase |
| A5CUC6 | DNA-directed RNA polymerase subunit beta' OS=Clavibacter michiganensis subsp. michiganensis (strain NCPPB 382) GN=rpoC PE=3 SV=1 | cmi03020 RNA polymerase |
| B0RB25 | DNA-directed RNA polymerase subunit beta OS=Clavibacter michiganensis subsp. sepedonicus (strain ATCC 33113 / DSM 20744 / JCM 9667 / LMG 2889 / C-1) GN=rpoB PE=3 SV=1 | cms03020 RNA polymerase |
| Q97EG9 | DNA-directed RNA polymerase subunit beta OS=Clostridium acetobutylicum (strain ATCC 824 / DSM 792 / JCM 1419 / LMG 5710 / VKM B-1787) GN=rpoB PE=3 SV=1 | cac03020 RNA polymerase |
| A6LPQ4 | DNA-directed RNA polymerase subunit beta OS=Clostridium beijerinckii (strain ATCC 51743 / NCIMB 8052) GN=rpoB PE=3 SV=1 | cbe03020 RNA polymerase |
| A6LPQ5 | DNA-directed RNA polymerase subunit beta' OS=Clostridium beijerinckii (strain ATCC 51743 / NCIMB 8052) GN=rpoC PE=3 SV=1 | cbe03020 RNA polymerase |
| B2UYA4 | DNA-directed RNA polymerase subunit beta' OS=Clostridium botulinum (strain Alaska E43 / Type E3) GN=rpoC PE=3 SV=1 | cbt03020 RNA polymerase |
| B1KSN3 | DNA-directed RNA polymerase subunit beta OS=Clostridium botulinum (strain Loch Maree / Type A3) GN=rpoB PE=3 SV=1 | cbl03020 RNA polymerase |
| B1IGG1 | DNA-directed RNA polymerase subunit beta' OS=Clostridium botulinum (strain Okra / Type B1) GN=rpoC PE=3 SV=1 | cbb03020 RNA polymerase |
| B9DYA1 | DNA-directed RNA polymerase subunit beta OS=Clostridium kluyveri (strain NBRC 12016) GN=rpoB PE=3 SV=1 | ckr03020 RNA polymerase |
| Q0SQD7 | DNA-directed RNA polymerase subunit beta' OS=Clostridium perfringens (strain SM101 / Type A) GN=rpoC PE=3 SV=1 | cpr03020 RNA polymerase |
| Q890N5 | DNA-directed RNA polymerase subunit beta' OS=Clostridium tetani (strain Massachusetts / E88) GN=rpoC PE=3 SV=2 | ctc03020 RNA polymerase |
| Q47UV9 | DNA-directed RNA polymerase subunit beta OS=Colwellia psychrerythraea (strain 34H / ATCC BAA-681) GN=rpoB PE=3 SV=1 | cps03020 RNA polymerase |
| C3PKN2 | DNA-directed RNA polymerase subunit beta' OS=Corynebacterium aurimucosum (strain ATCC 700975 / DSM 44827 / CN-1) GN=rpoC PE=3 SV=1 | car03020 RNA polymerase |
| Q6NJF6 | DNA-directed RNA polymerase subunit beta' OS=Corynebacterium diphtheriae (strain ATCC 700971 / NCTC 13129 / Biotype gravis) GN=rpoC PE=3 SV=1 | cdi03020 RNA polymerase |
| Q8FS96 | DNA-directed RNA polymerase subunit beta' OS=Corynebacterium efficiens (strain DSM 44549 / YS-314 / AJ 12310 / JCM 11189 / NBRC 100395) GN=rpoC PE=3 SV=1 | cef03020 RNA polymerase |
| A4QBG2 | DNA-directed RNA polymerase subunit beta OS=Corynebacterium glutamicum (strain R) GN=rpoB PE=3 SV=1 | cgt03020 RNA polymerase |
| Q4JT33 | DNA-directed RNA polymerase subunit beta' OS=Corynebacterium jeikeium (strain K411) GN=rpoC PE=3 SV=1 | cjk03020 RNA polymerase |
| C4LL70 | DNA-directed RNA polymerase subunit beta' OS=Corynebacterium kroppenstedtii (strain DSM 44385 / JCM 11950 / CIP 105744 / CCUG 35717) GN=rpoC PE=3 SV=1 | ckp03020 RNA polymerase |
| A9NAL6 | DNA-directed RNA polymerase subunit beta' OS=Coxiella burnetii (strain RSA 331 / Henzerling II) GN=rpoC PE=3 SV=1 | cbs03020 RNA polymerase |
| Q1LI20 | DNA-directed RNA polymerase subunit beta OS=Cupriavidus metallidurans (strain ATCC 43123 / DSM 2839 / NBRC 102507 / CH34) GN=rpoB PE=3 SV=1 | rme03020 RNA polymerase |
| Q1LI21 | DNA-directed RNA polymerase subunit beta' OS=Cupriavidus metallidurans (strain ATCC 43123 / DSM 2839 / NBRC 102507 / CH34) GN=rpoC PE=3 SV=1 | rme03020 RNA polymerase |
| Q0K607 | DNA-directed RNA polymerase subunit beta' OS=Cupriavidus necator (strain ATCC 17699 / H16 / DSM 428 / Stanier 337) GN=rpoC PE=3 SV=1 | reh03020 RNA polymerase |
| A5FRK5 | DNA-directed RNA polymerase subunit beta OS=Dehalococcoides mccartyi (strain ATCC BAA-2100 / JCM 16839 / KCTC 5957 / BAV1) GN=rpoB PE=3 SV=2 | deb03020 RNA polymerase |
| A5FRK6 | DNA-directed RNA polymerase subunit beta' OS=Dehalococcoides mccartyi (strain ATCC BAA-2100 / JCM 16839 / KCTC 5957 / BAV1) GN=rpoC PE=3 SV=2 | deb03020 RNA polymerase |
| Q1J0P6 | DNA-directed RNA polymerase subunit beta OS=Deinococcus geothermalis (strain DSM 11300) GN=rpoB PE=3 SV=1 | dge03020 RNA polymerase |
| Q9RVV9 | DNA-directed RNA polymerase subunit beta OS=Deinococcus radiodurans (strain ATCC 13939 / DSM 20539 / JCM 16871 / LMG 4051 / NBRC 15346 / NCIMB 9279 / R1 / VKM B-1422) GN=rpoB PE=3 SV=2 | dra03020 RNA polymerase |
| B8FEU1 | DNA-directed RNA polymerase subunit beta OS=Desulfatibacillum alkenivorans (strain AK-01) GN=rpoB PE=3 SV=1 | dal03020 RNA polymerase |
| C0Q9X9 | DNA-directed RNA polymerase subunit beta OS=Desulfobacterium autotrophicum (strain ATCC 43914 / DSM 3382 / HRM2) GN=rpoB PE=3 SV=1 | dat03020 RNA polymerase |
| A8ZV51 | DNA-directed RNA polymerase subunit beta OS=Desulfococcus oleovorans (strain DSM 6200 / Hxd3) GN=rpoB PE=3 SV=1 | dol03020 RNA polymerase |
| A8ZV52 | DNA-directed RNA polymerase subunit beta' OS=Desulfococcus oleovorans (strain DSM 6200 / Hxd3) GN=rpoC PE=3 SV=1 | dol03020 RNA polymerase |
| B1I1M9 | DNA-directed RNA polymerase subunit beta' OS=Desulforudis audaxviator (strain MP104C) GN=rpoC PE=3 SV=1 | dau03020 RNA polymerase |
| A4J104 | DNA-directed RNA polymerase subunit beta' OS=Desulfotomaculum reducens (strain MI-1) GN=rpoC PE=3 SV=1 | drm03020 RNA polymerase |
| Q30X05 | DNA-directed RNA polymerase subunit beta OS=Desulfovibrio alaskensis (strain G20) GN=rpoB PE=3 SV=1 | dde03020 RNA polymerase |
| B8J1A8 | DNA-directed RNA polymerase subunit beta OS=Desulfovibrio desulfuricans (strain ATCC 27774 / DSM 6949) GN=rpoB PE=3 SV=1 | dds03020 RNA polymerase |
| C4XIN9 | DNA-directed RNA polymerase subunit beta OS=Desulfovibrio magneticus (strain ATCC 700980 / DSM 13731 / RS-1) GN=rpoB PE=3 SV=1 | dma03020 RNA polymerase |
| C6C179 | DNA-directed RNA polymerase subunit beta OS=Desulfovibrio salexigens (strain ATCC 14822 / DSM 2638 / NCIB 8403 / VKM B-1763) GN=rpoB PE=3 SV=1 | dsa03020 RNA polymerase |
| Q727C7 | DNA-directed RNA polymerase subunit beta OS=Desulfovibrio vulgaris (strain Hildenborough / ATCC 29579 / NCIMB 8303) GN=rpoB PE=3 SV=1 | dvu03020 RNA polymerase |
| Q727C6 | DNA-directed RNA polymerase subunit beta' OS=Desulfovibrio vulgaris (strain Hildenborough / ATCC 29579 / NCIMB 8303) GN=rpoC PE=3 SV=1 | dvu03020 RNA polymerase |
| A5EX70 | DNA-directed RNA polymerase subunit beta OS=Dichelobacter nodosus (strain VCS1703A) GN=rpoB PE=3 SV=1 | dno03020 RNA polymerase |
| A5EX69 | DNA-directed RNA polymerase subunit beta' OS=Dichelobacter nodosus (strain VCS1703A) GN=rpoC PE=3 SV=1 | dno03020 RNA polymerase |
| A8LM40 | DNA-directed RNA polymerase subunit beta OS=Dinoroseobacter shibae (strain DSM 16493 / NCIMB 14021 / DFL 12) GN=rpoB PE=3 SV=1 | dsh03020 RNA polymerase |
| Q3YST4 | DNA-directed RNA polymerase subunit beta' OS=Ehrlichia canis (strain Jake) GN=rpoC PE=3 SV=1 | ecn03020 RNA polymerase |
| Q8KWX2 | DNA-directed RNA polymerase subunit beta OS=Ehrlichia chaffeensis (strain ATCC CRL-10679 / Arkansas) GN=rpoB PE=3 SV=2 | ech03020 RNA polymerase |
| Q5HC04 | DNA-directed RNA polymerase subunit beta' OS=Ehrlichia ruminantium (strain Welgevonden) GN=rpoC PE=3 SV=1 | eru03020 RNA polymerase |
| B2KEN0 | DNA-directed RNA polymerase subunit beta' OS=Elusimicrobium minutum (strain Pei191) GN=rpoC PE=3 SV=1 | emi03020 RNA polymerase |
| Q8GCR3 | DNA-directed RNA polymerase subunit beta OS=Enterococcus faecium GN=rpoB PE=3 SV=1 | RNA polymerase |
| B2VG96 | DNA-directed RNA polymerase subunit beta OS=Erwinia tasmaniensis (strain DSM 17950 / CIP 109463 / Et1/99) GN=rpoB PE=3 SV=1 | eta03020 RNA polymerase |
| A8A787 | DNA-directed RNA polymerase subunit beta' OS=Escherichia coli O9:H4 (strain HS) GN=rpoC PE=3 SV=1 | ecx03020 RNA polymerase |
| B1YGU3 | DNA-directed RNA polymerase subunit beta' OS=Exiguobacterium sibiricum (strain DSM 17290 / JCM 13490 / 255-15) GN=rpoC PE=3 SV=1 | esi03020 RNA polymerase |
| A7HNY2 | DNA-directed RNA polymerase subunit beta OS=Fervidobacterium nodosum (strain ATCC 35602 / DSM 5306 / Rt17-B1) GN=rpoB PE=3 SV=1 | fno03020 RNA polymerase |
| A7HNY1 | DNA-directed RNA polymerase subunit beta' OS=Fervidobacterium nodosum (strain ATCC 35602 / DSM 5306 / Rt17-B1) GN=rpoC PE=3 SV=1 | fno03020 RNA polymerase |
| A5FIJ3 | DNA-directed RNA polymerase subunit beta OS=Flavobacterium johnsoniae (strain ATCC 17061 / DSM 2064 / UW101) GN=rpoB PE=3 SV=1 | fjo03020 RNA polymerase |
| A6GYT9 | DNA-directed RNA polymerase subunit beta' OS=Flavobacterium psychrophilum (strain JIP02/86 / ATCC 49511) GN=rpoC PE=3 SV=1 | fps03020 RNA polymerase |
| Q0BKC6 | DNA-directed RNA polymerase subunit beta' OS=Francisella tularensis subsp. holarctica (strain OSU18) GN=rpoC PE=3 SV=1 | fth03020 RNA polymerase |
| A8LC63 | DNA-directed RNA polymerase subunit beta' OS=Frankia sp. (strain EAN1pec) GN=rpoC PE=3 SV=1 | fre03020 RNA polymerase |
| Q8RHI6 | DNA-directed RNA polymerase subunit beta OS=Fusobacterium nucleatum subsp. nucleatum (strain ATCC 25586 / CIP 101130 / JCM 8532 / LMG 13131) GN=rpoB PE=3 SV=1 | fnu03020 RNA polymerase |
| Q8RHI7 | DNA-directed RNA polymerase subunit beta' OS=Fusobacterium nucleatum subsp. nucleatum (strain ATCC 25586 / CIP 101130 / JCM 8532 / LMG 13131) GN=rpoC PE=3 SV=1 | fnu03020 RNA polymerase |
| A4IJI2 | DNA-directed RNA polymerase subunit beta' OS=Geobacillus thermodenitrificans (strain NG80-2) GN=rpoC PE=3 SV=2 | gtn03020 RNA polymerase |
| B5EFP3 | DNA-directed RNA polymerase subunit beta OS=Geobacter bemidjiensis (strain Bem / ATCC BAA-1014 / DSM 16622) GN=rpoB PE=3 SV=1 | gbm03020  RNA polymerase |
| B5EFP4 | DNA-directed RNA polymerase subunit beta' OS=Geobacter bemidjiensis (strain Bem / ATCC BAA-1014 / DSM 16622) GN=rpoC PE=3 SV=1 | gbm03020 RNA polymerase |
| B9M6V2 | DNA-directed RNA polymerase subunit beta OS=Geobacter daltonii (strain DSM 22248 / JCM 15807 / FRC-32) GN=rpoB PE=3 SV=1 | geo03020 RNA polymerase |
| B9M6V1 | DNA-directed RNA polymerase subunit beta' OS=Geobacter daltonii (strain DSM 22248 / JCM 15807 / FRC-32) GN=rpoC PE=3 SV=1 | geo03020 RNA polymerase |
| B3E7S8 | DNA-directed RNA polymerase subunit beta OS=Geobacter lovleyi (strain ATCC BAA-1151 / DSM 17278 / SZ) GN=rpoB PE=3 SV=1 | glo03020 RNA polymerase |
| Q39Y13 | DNA-directed RNA polymerase subunit beta OS=Geobacter metallireducens (strain GS-15 / ATCC 53774 / DSM 7210) GN=rpoB PE=3 SV=1 | gme03020 RNA polymerase |
| Q748Y6 | DNA-directed RNA polymerase subunit beta OS=Geobacter sulfurreducens (strain ATCC 51573 / DSM 12127 / PCA) GN=rpoB PE=3 SV=1 | gsu03020 RNA polymerase |
| A5GAY2 | DNA-directed RNA polymerase subunit beta' OS=Geobacter uraniireducens (strain Rf4) GN=rpoC PE=3 SV=1 | gur03020 RNA polymerase |
| A9H3S0 | DNA-directed RNA polymerase subunit beta' OS=Gluconacetobacter diazotrophicus (strain ATCC 49037 / DSM 5601 / PAl5) GN=rpoC PE=3 SV=1 | gdi03020 RNA polymerase |
| Q5FTX8 | DNA-directed RNA polymerase subunit beta' OS=Gluconobacter oxydans (strain 621H) GN=rpoC PE=3 SV=1 | gox03020 RNA polymerase |
| A0M3Y8 | DNA-directed RNA polymerase subunit beta' OS=Gramella forsetii (strain KT0803) GN=rpoC PE=3 SV=1 | gfo03020 RNA polymerase |
| Q0BUQ6 | DNA-directed RNA polymerase subunit beta OS=Granulibacter bethesdensis (strain ATCC BAA-1260 / CGDNIH1) GN=rpoB PE=3 SV=1 | gbe03020 RNA polymerase |
| Q0BUQ5 | DNA-directed RNA polymerase subunit beta' OS=Granulibacter bethesdensis (strain ATCC BAA-1260 / CGDNIH1) GN=rpoC PE=3 SV=1 | gbe03020 RNA polymerase |
| Q7VKL7 | DNA-directed RNA polymerase subunit beta OS=Haemophilus ducreyi (strain 35000HP / ATCC 700724) GN=rpoB PE=3 SV=1 | hdu03020 RNA polymerase |
| Q0I5B8 | DNA-directed RNA polymerase subunit beta' OS=Haemophilus somnus (strain 129Pt) GN=rpoC PE=3 SV=1 | hso03020 RNA polymerase |
| Q2S906 | DNA-directed RNA polymerase subunit beta' OS=Hahella chejuensis (strain KCTC 2396) GN=rpoC PE=3 SV=1 | hch03020 RNA polymerase |
| A1WVC8 | DNA-directed RNA polymerase subunit beta' OS=Halorhodospira halophila (strain DSM 244 / SL1) GN=rpoC PE=3 SV=2 | hha03020 RNA polymerase |
| C4K4F1 | DNA-directed RNA polymerase subunit beta OS=Hamiltonella defensa subsp. Acyrthosiphon pisum (strain 5AT) GN=rpoB PE=3 SV=1 | hde03020 RNA polymerase |
| B0TC49 | DNA-directed RNA polymerase subunit beta' OS=Heliobacterium modesticaldum (strain ATCC 51547 / Ice1) GN=rpoC PE=3 SV=1 | hmo03020 RNA polymerase |
| A4G9U5 | DNA-directed RNA polymerase subunit beta OS=Herminiimonas arsenicoxydans GN=rpoB PE=3 SV=1 | har03020 RNA polymerase |
| A4G9U4 | DNA-directed RNA polymerase subunit beta' OS=Herminiimonas arsenicoxydans GN=rpoC PE=3 SV=1 | har03020 RNA polymerase |
| A9B6J3 | DNA-directed RNA polymerase subunit beta OS=Herpetosiphon aurantiacus (strain ATCC 23779 / DSM 785) GN=rpoB PE=3 SV=1 | hau03020 RNA polymerase |
| A9B6J1 | DNA-directed RNA polymerase subunit beta' OS=Herpetosiphon aurantiacus (strain ATCC 23779 / DSM 785) GN=rpoC PE=3 SV=1 | hau03020 RNA polymerase |
| B4U738 | DNA-directed RNA polymerase subunit beta' OS=Hydrogenobaculum sp. (strain Y04AAS1) GN=rpoC PE=3 SV=1 | hya03020 RNA polymerase |
| Q0BYA7 | DNA-directed RNA polymerase subunit beta OS=Hyphomonas neptunium (strain ATCC 15444) GN=rpoB PE=3 SV=1 | hne03020 RNA polymerase |
| Q0BYA8 | DNA-directed RNA polymerase subunit beta' OS=Hyphomonas neptunium (strain ATCC 15444) GN=rpoC PE=3 SV=1 | RNA polymerase |
| Q5QWA4 | DNA-directed RNA polymerase subunit beta' OS=Idiomarina loihiensis (strain ATCC BAA-735 / DSM 15497 / L2-TR) GN=rpoC PE=3 SV=1 | ilo03020 RNA polymerase |
| Q28UX7 | DNA-directed RNA polymerase subunit beta OS=Jannaschia sp. (strain CCS1) GN=rpoB PE=3 SV=2 | jan03020 RNA polymerase |
| A6TGP1 | DNA-directed RNA polymerase subunit beta' OS=Klebsiella pneumoniae subsp. pneumoniae (strain ATCC 700721 / MGH 78578) GN=rpoC PE=3 SV=1 | kpn03020 RNA polymerase |
| Q1IHH4 | DNA-directed RNA polymerase subunit beta OS=Koribacter versatilis (strain Ellin345) GN=rpoB PE=3 SV=1 | aba03020 RNA polymerase |
| Q1IHH5 | DNA-directed RNA polymerase subunit beta' OS=Koribacter versatilis (strain Ellin345) GN=rpoC PE=3 SV=2 | aba03020 RNA polymerase |
| C5CGD9 | DNA-directed RNA polymerase subunit beta OS=Kosmotoga olearia (strain TBF 19.5.1) GN=rpoB PE=3 SV=1 | kol03020 RNA polymerase |
| Q03PV0 | DNA-directed RNA polymerase subunit beta' OS=Lactobacillus brevis (strain ATCC 367 / JCM 1170) GN=rpoC PE=3 SV=1 | lbr03020 RNA polymerase |
| B3WAM8 | DNA-directed RNA polymerase subunit beta OS=Lactobacillus casei (strain BL23) GN=rpoB PE=3 SV=1 | lcb03020 RNA polymerase |
| Q046D1 | DNA-directed RNA polymerase subunit beta' OS=Lactobacillus gasseri (strain ATCC 33323 / DSM 20243 / JCM 1131 / NCIMB 11718 / AM63) GN=rpoC PE=3 SV=1 | lga03020 RNA polymerase |
| B2G8Y5 | DNA-directed RNA polymerase subunit beta' OS=Lactobacillus reuteri (strain JCM 1112) GN=rpoC PE=3 SV=1 | lrf03020 RNA polymerase |
| Q1WVA4 | DNA-directed RNA polymerase subunit beta' OS=Lactobacillus salivarius (strain UCC118) GN=rpoC PE=3 SV=1 | lsl03020 RNA polymerase |
| Q1MPW8 | DNA-directed RNA polymerase subunit beta OS=Lawsonia intracellularis (strain PHE/MN1-00) GN=rpoB PE=3 SV=1 | lip03020 RNA polymerase |
| Q1MPW9 | DNA-directed RNA polymerase subunit beta' OS=Lawsonia intracellularis (strain PHE/MN1-00) GN=rpoC PE=3 SV=1 | lip03020 RNA polymerase |
| A5IHS1 | DNA-directed RNA polymerase subunit beta OS=Legionella pneumophila (strain Corby) GN=rpoB PE=3 SV=1 | lpc03020 RNA polymerase |
| A5IHS0 | DNA-directed RNA polymerase subunit beta' OS=Legionella pneumophila (strain Corby) GN=rpoC PE=3 SV=2 | lpc03020 RNA polymerase |
| B0SAG1 | DNA-directed RNA polymerase subunit beta OS=Leptospira biflexa serovar Patoc (strain Patoc 1 / Ames) GN=rpoB PE=3 SV=1 | lbf03020 RNA polymerase |
| Q04QI9 | DNA-directed RNA polymerase subunit beta OS=Leptospira borgpetersenii serovar Hardjo-bovis (strain JB197) GN=rpoB PE=3 SV=1 | lbj03020 RNA polymerase |
| B1Y7H2 | DNA-directed RNA polymerase subunit beta' OS=Leptothrix cholodnii (strain ATCC 51168 / LMG 8142 / SP-6) GN=rpoC PE=3 SV=1 | lch03020 RNA polymerase |
| B1MVW7 | DNA-directed RNA polymerase subunit beta' OS=Leuconostoc citreum (strain KM20) GN=rpoC PE=3 SV=1 | lci03020 RNA polymerase |
| B8DEY8 | DNA-directed RNA polymerase subunit beta OS=Listeria monocytogenes serotype 4a (strain HCC23) GN=rpoB PE=3 SV=1 | lmh03020 RNA polymerase |
| Q8YA96 | DNA-directed RNA polymerase subunit beta' OS=Listeria monocytogenes serovar 1/2a (strain ATCC BAA-679 / EGD-e) GN=rpoC PE=3 SV=1 | lmo03020 RNA polymerase |
| B1HMZ5 | DNA-directed RNA polymerase subunit beta' OS=Lysinibacillus sphaericus (strain C3-41) GN=rpoC PE=3 SV=2 | lsp03020 RNA polymerase |
| B9E8Q5 | DNA-directed RNA polymerase subunit beta OS=Macrococcus caseolyticus (strain JCSC5402) GN=rpoB PE=3 SV=1 | mcl03020 RNA polymerase |
| A0L5W7 | DNA-directed RNA polymerase subunit beta' OS=Magnetococcus marinus (strain ATCC BAA-1437 / JCM 17883 / MC-1) GN=rpoC PE=3 SV=1 | mgm03020 RNA polymerase |
| Q0ANP3 | DNA-directed RNA polymerase subunit beta OS=Maricaulis maris (strain MCS10) GN=rpoB PE=3 SV=1 | mmr03020 RNA polymerase |
| A1TYJ1 | DNA-directed RNA polymerase subunit beta' OS=Marinobacter hydrocarbonoclasticus (strain ATCC 700491 / DSM 11845 / VT8) GN=rpoC PE=3 SV=1 | maq03020 RNA polymerase |
| A6W398 | DNA-directed RNA polymerase subunit beta' OS=Marinomonas sp. (strain MWYL1) GN=rpoC PE=3 SV=1 | mmw03020 RNA polymerase |
| B3E163 | DNA-directed RNA polymerase subunit beta OS=Methylacidiphilum infernorum (isolate V4) GN=rpoB PE=3 SV=1 | min03020 RNA polymerase |
| A2SLG5 | DNA-directed RNA polymerase subunit beta OS=Methylibium petroleiphilum (strain ATCC BAA-1232 / LMG 22953 / PM1) GN=rpoB PE=3 SV=1 | mpt03020 RNA polymerase |
| A9W8N8 | DNA-directed RNA polymerase subunit beta OS=Methylobacterium extorquens (strain PA1) GN=rpoB PE=3 SV=1 | mex03020 RNA polymerase |
| Q60A05 | DNA-directed RNA polymerase subunit beta' OS=Methylococcus capsulatus (strain ATCC 33009 / NCIMB 11132 / Bath) GN=rpoC PE=3 SV=1 | mca03020 RNA polymerase |
| Q2RFP0 | DNA-directed RNA polymerase subunit beta' OS=Moorella thermoacetica (strain ATCC 39073 / JCM 9320) GN=rpoC PE=3 SV=1 | mta03020 RNA polymerase |
| A5U052 | DNA-directed RNA polymerase subunit beta OS=Mycobacterium tuberculosis (strain ATCC 25177 / H37Ra) GN=rpoB PE=3 SV=2 | mra03020 RNA polymerase |
| P47715 | DNA-directed RNA polymerase subunit beta OS=Mycoplasma gallisepticum (strain R(low / passage 15 / clone 2)) GN=rpoB PE=3 SV=2 | mga03020 RNA polymerase |
| Q4A7A9 | DNA-directed RNA polymerase subunit beta OS=Mycoplasma hyopneumoniae (strain 7448) GN=rpoB PE=3 SV=1 | mhp03020 RNA polymerase |
| Q4A5S7 | DNA-directed RNA polymerase subunit beta OS=Mycoplasma synoviae (strain 53) GN=rpoB PE=3 SV=2 | msy03020 RNA polymerase |
| Q1D7U2 | DNA-directed RNA polymerase subunit beta' OS=Myxococcus xanthus (strain DK 1622) GN=rpoC PE=3 SV=1 | mxa03020 RNA polymerase |
| P57009 | DNA-directed RNA polymerase subunit beta OS=Neisseria meningitidis serogroup A / serotype 4A (strain Z2491) GN=rpoB PE=3 SV=1 | nma03020 RNA polymerase |
| Q9JX03 | DNA-directed RNA polymerase subunit beta' OS=Neisseria meningitidis serogroup A / serotype 4A (strain Z2491) GN=rpoC PE=3 SV=1 | nma03020 RNA polymerase |
| Q2GD91 | DNA-directed RNA polymerase subunit beta' OS=Neorickettsia sennetsu (strain ATCC VR-367 / Miyayama) GN=rpoC PE=3 SV=1 | nse03020 RNA polymerase |
| A6Q1M3 | DNA-directed RNA polymerase subunit beta OS=Nitratiruptor sp. (strain SB155-2) GN=rpoB PE=3 SV=1 | nis03020 RNA polymerase |
| A6Q1M4 | DNA-directed RNA polymerase subunit beta' OS=Nitratiruptor sp. (strain SB155-2) GN=rpoC PE=3 SV=1 | nis03020 RNA polymerase |
| Q1QN43 | DNA-directed RNA polymerase subunit beta' OS=Nitrobacter hamburgensis (strain DSM 10229 / NCIMB 13809 / X14) GN=rpoC PE=3 SV=1 | nha03020 RNA polymerase |
| Q3SSX9 | DNA-directed RNA polymerase subunit beta' OS=Nitrobacter winogradskyi (strain ATCC 25391 / DSM 10237 / CIP 104748 / NCIMB 11846 / Nb-255) GN=rpoC PE=3 SV=1 | nwi03020 RNA polymerase |
| Q3J8Q7 | DNA-directed RNA polymerase subunit beta OS=Nitrosococcus oceani (strain ATCC 19707 / BCRC 17464 / NCIMB 11848 / C-107) GN=rpoB PE=3 SV=1 | noc03020 RNA polymerase |
| Q0AF53 | DNA-directed RNA polymerase subunit beta OS=Nitrosomonas eutropha (strain C91) GN=rpoB PE=3 SV=1 | net03020 RNA polymerase |
| Q2YB05 | DNA-directed RNA polymerase subunit beta OS=Nitrosospira multiformis (strain ATCC 25196 / NCIMB 11849 / C 71) GN=rpoB PE=3 SV=1 | nmu03020 RNA polymerase |
| Q2YB04 | DNA-directed RNA polymerase subunit beta' OS=Nitrosospira multiformis (strain ATCC 25196 / NCIMB 11849 / C 71) GN=rpoC PE=3 SV=1 | nmu03020 RNA polymerase |
| A1SEK1 | DNA-directed RNA polymerase subunit beta OS=Nocardioides sp. (strain ATCC BAA-499 / JS614) GN=rpoB PE=3 SV=1 | nca03020 RNA polymerase |
| Q2GCD7 | DNA-directed RNA polymerase subunit beta OS=Novosphingobium aromaticivorans (strain ATCC 700278 / DSM 12444 / CIP 105152 / NBRC 16084 / F199) GN=rpoB PE=3 SV=1 | nar03020 RNA polymerase |
| Q2GCD8 | DNA-directed RNA polymerase subunit beta' OS=Novosphingobium aromaticivorans (strain ATCC 700278 / DSM 12444 / CIP 105152 / NBRC 16084 / F199) GN=rpoC PE=3 SV=1 | nar03020 RNA polymerase |
| Q8ETY8 | DNA-directed RNA polymerase subunit beta OS=Oceanobacillus iheyensis (strain DSM 14371 / CIP 107618 / JCM 11309 / KCTC 3954 / HTE831) GN=rpoB PE=3 SV=1 | oih03020 RNA polymerase |
| Q04E86 | DNA-directed RNA polymerase subunit beta' OS=Oenococcus oeni (strain ATCC BAA-331 / PSU-1) GN=rpoC PE=3 SV=1 | ooe03020 RNA polymerase |
| B6JES1 | DNA-directed RNA polymerase subunit beta' OS=Oligotropha carboxidovorans (strain ATCC 49405 / DSM 1227 / KCTC 32145 / OM5) GN=rpoC PE=3 SV=1 | oca03020 RNA polymerase |
| B1ZPB7 | DNA-directed RNA polymerase subunit beta' OS=Opitutus terrae (strain DSM 11246 / JCM 15787 / PB90-1) GN=rpoC PE=3 SV=1 | ote03020 RNA polymerase |
| A1B015 | DNA-directed RNA polymerase subunit beta OS=Paracoccus denitrificans (strain Pd 1222) GN=rpoB PE=3 SV=1 | pde03020 RNA polymerase |
| A1B017 | DNA-directed RNA polymerase subunit beta' OS=Paracoccus denitrificans (strain Pd 1222) GN=rpoC PE=3 SV=1 | pde03020 RNA polymerase |
| A7HWQ4 | DNA-directed RNA polymerase subunit beta OS=Parvibaculum lavamentivorans (strain DS-1 / DSM 13023 / NCIMB 13966) GN=rpoB PE=3 SV=1 | pla03020 RNA polymerase |
| Q9CK91 | DNA-directed RNA polymerase subunit beta OS=Pasteurella multocida (strain Pm70) GN=rpoB PE=3 SV=1 | pmu03020 RNA polymerase |
| Q4FLL3 | DNA-directed RNA polymerase subunit beta' OS=Pelagibacter ubique (strain HTCC1062) GN=rpoC PE=3 SV=1 | pub03020 RNA polymerase |
| Q3A6Q4 | DNA-directed RNA polymerase subunit beta OS=Pelobacter carbinolicus (strain DSM 2380 / NBRC 103641 / GraBd1) GN=rpoB PE=3 SV=1 | pca03020 RNA polymerase |
| A1ALT4 | DNA-directed RNA polymerase subunit beta OS=Pelobacter propionicus (strain DSM 2379 / NBRC 103807 / OttBd1) GN=rpoB PE=3 SV=1 | ppd03020 RNA polymerase |
| B4SG10 | DNA-directed RNA polymerase subunit beta' OS=Pelodictyon phaeoclathratiforme (strain DSM 5477 / BU-1) GN=rpoC PE=3 SV=1 | pth03020 RNA polymerase |
| A5D5I2 | DNA-directed RNA polymerase subunit beta OS=Pelotomaculum thermopropionicum (strain DSM 13744 / JCM 10971 / SI) GN=rpoB PE=3 SV=1 | pph03020 RNA polymerase |
| Q18CF3 | DNA-directed RNA polymerase subunit beta' OS=Peptoclostridium difficile (strain 630) GN=rpoC PE=3 SV=1 | pth03020 RNA polymerase |
| A9BF32 | DNA-directed RNA polymerase subunit beta' OS=Petrotoga mobilis (strain DSM 10674 / SJ95) GN=rpoC PE=3 SV=1 | pmo03020 RNA polymerase |
| B4R8K5 | DNA-directed RNA polymerase subunit beta OS=Phenylobacterium zucineum (strain HLK1) GN=rpoB PE=3 SV=1 | pzu03020 RNA polymerase |
| Q6LLW3 | DNA-directed RNA polymerase subunit beta' OS=Photobacterium profundum (strain SS9) GN=rpoC PE=3 SV=2 | ppr03020 RNA polymerase |
| Q7N9A4 | DNA-directed RNA polymerase subunit beta OS=Photorhabdus luminescens subsp. laumondii (strain DSM 15139 / CIP 105565 / TT01) GN=rpoB PE=3 SV=1 | plu03020 RNA polymerase |
| B1VAM6 | DNA-directed RNA polymerase subunit beta OS=Phytoplasma australiense GN=rpoB PE=3 SV=1 | pal03020 RNA polymerase |
| A1VTF7 | DNA-directed RNA polymerase subunit beta' OS=Polaromonas naphthalenivorans (strain CJ2) GN=rpoC PE=3 SV=1 | pna03020 RNA polymerase |
| B1XSP4 | DNA-directed RNA polymerase subunit beta' OS=Polynucleobacter necessarius subsp. necessarius (strain STIR1) GN=rpoC PE=3 SV=1 | pne03020 RNA polymerase |
| Q7MX26 | DNA-directed RNA polymerase subunit beta' OS=Porphyromonas gingivalis (strain ATCC BAA-308 / W83) GN=rpoC PE=3 SV=1 | pgi03020 RNA polymerase |
| A2C6S8 | DNA-directed RNA polymerase subunit beta OS=Prochlorococcus marinus (strain MIT 9303) GN=rpoB PE=3 SV=1 | pmf03020 RNA polymerase |
| A2BYL1 | DNA-directed RNA polymerase subunit beta OS=Prochlorococcus marinus (strain MIT 9515) GN=rpoB PE=3 SV=1 | pmc03020 RNA polymerase |
| Q6MDM0 | DNA-directed RNA polymerase subunit beta' OS=Protochlamydia amoebophila (strain UWE25) GN=rpoC PE=3 SV=1 | pcu03020 RNA polymerase |
| Q15YB1 | DNA-directed RNA polymerase subunit beta OS=Pseudoalteromonas atlantica (strain T6c / ATCC BAA-1087) GN=rpoB PE=3 SV=1 | pat03020 RNA polymerase |
| Q15YB0 | DNA-directed RNA polymerase subunit beta' OS=Pseudoalteromonas atlantica (strain T6c / ATCC BAA-1087) GN=rpoC PE=3 SV=1 | pat03020  RNA polymerase |
| Q3ILP9 | DNA-directed RNA polymerase subunit beta OS=Pseudoalteromonas haloplanktis (strain TAC 125) GN=rpoB PE=3 SV=1 | RNA polymerase |
| B1JDX1 | DNA-directed RNA polymerase subunit beta OS=Pseudomonas putida (strain W619) GN=rpoB PE=3 SV=1 | ppw03020 RNA polymerase |
| P19175 | DNA-directed RNA polymerase subunit beta OS=Pseudomonas putida GN=rpoB PE=3 SV=2 | RNA polymerase |
| P19176 | DNA-directed RNA polymerase subunit beta' OS=Pseudomonas putida GN=rpoC PE=3 SV=3 | RNA polymerase |
| A4VHM4 | DNA-directed RNA polymerase subunit beta' OS=Pseudomonas stutzeri (strain A1501) GN=rpoC PE=3 SV=1 | psa03020 RNA polymerase |
| A8F4G0 | DNA-directed RNA polymerase subunit beta OS=Pseudothermotoga lettingae (strain ATCC BAA-301 / DSM 14385 / NBRC 107922 / TMO) GN=rpoB PE=3 SV=1 | tle03020 RNA polymerase |
| A8F4G1 | DNA-directed RNA polymerase subunit beta' OS=Pseudothermotoga lettingae (strain ATCC BAA-301 / DSM 14385 / NBRC 107922 / TMO) GN=rpoC PE=3 SV=1 | tle03020 RNA polymerase |
| Q4FQH3 | DNA-directed RNA polymerase subunit beta OS=Psychrobacter arcticus (strain DSM 17307 / 273-4) GN=rpoB PE=3 SV=1 | par03020 RNA polymerase |
| Q1Q8Q0 | DNA-directed RNA polymerase subunit beta' OS=Psychrobacter cryohalolentis (strain K5) GN=rpoC PE=3 SV=1 | pcr03020 RNA polymerase |
| A5WH34 | DNA-directed RNA polymerase subunit beta' OS=Psychrobacter sp. (strain PRwf-1) GN=rpoC PE=3 SV=1 | prw03020 RNA polymerase |
| A1T065 | DNA-directed RNA polymerase subunit beta OS=Psychromonas ingrahamii (strain 37) GN=rpoB PE=3 SV=1 | pin03020 RNA polymerase |
| A1T064 | DNA-directed RNA polymerase subunit beta' OS=Psychromonas ingrahamii (strain 37) GN=rpoC PE=3 SV=1 | pin03020 RNA polymerase |
| A9WSY0 | DNA-directed RNA polymerase subunit beta OS=Renibacterium salmoninarum (strain ATCC 33209 / DSM 20767 / JCM 11484 / NBRC 15589 / NCIMB 2235) GN=rpoB PE=3 SV=1 | rsa03020 RNA polymerase |
| Q98N66 | DNA-directed RNA polymerase subunit beta OS=Rhizobium loti (strain MAFF303099) GN=rpoB PE=3 SV=1 | mlo03020 RNA polymerase |
| Q98N65 | DNA-directed RNA polymerase subunit beta' OS=Rhizobium loti (strain MAFF303099) GN=rpoC PE=3 SV=1 | mlo03020 RNA polymerase |
| Q3J5S9 | DNA-directed RNA polymerase subunit beta' OS=Rhodobacter sphaeroides (strain ATCC 17023 / 2.4.1 / NCIB 8253 / DSM 158) GN=rpoC PE=3 SV=1 | rsp03020 RNA polymerase |
| C1AYV9 | DNA-directed RNA polymerase subunit beta OS=Rhodococcus opacus (strain B4) GN=rpoB PE=3 SV=1 | rop03020 RNA polymerase |
| Q211D8 | DNA-directed RNA polymerase subunit beta OS=Rhodopseudomonas palustris (strain BisB18) GN=rpoB PE=3 SV=1 | rpc03020 RNA polymerase |
| Q134R8 | DNA-directed RNA polymerase subunit beta OS=Rhodopseudomonas palustris (strain BisB5) GN=rpoB PE=3 SV=1 | rpd03020 RNA polymerase |
| Q134R9 | DNA-directed RNA polymerase subunit beta' OS=Rhodopseudomonas palustris (strain BisB5) GN=rpoC PE=3 SV=1 | rpd03020 RNA polymerase |
| B3QBZ9 | DNA-directed RNA polymerase subunit beta' OS=Rhodopseudomonas palustris (strain TIE-1) GN=rpoC PE=3 SV=1 | rpt03020 RNA polymerase |
| B6IRP6 | DNA-directed RNA polymerase subunit beta OS=Rhodospirillum centenum (strain ATCC 51521 / SW) GN=rpoB PE=3 SV=1 | rce03020 RNA polymerase |
| A8GMA8 | DNA-directed RNA polymerase subunit beta' OS=Rickettsia akari (strain Hartford) GN=rpoC PE=3 SV=1 | rak03020 RNA polymerase |
| Q4UKD4 | DNA-directed RNA polymerase subunit beta OS=Rickettsia felis (strain ATCC VR-1525 / URRWXCal2) GN=rpoB PE=3 SV=1 | rfe03020 RNA polymerase |
| Q68XM7 | DNA-directed RNA polymerase subunit beta' OS=Rickettsia typhi (strain ATCC VR-144 / Wilmington) GN=rpoC PE=3 SV=1 | rty03020 RNA polymerase |
| A7NJM0 | DNA-directed RNA polymerase subunit beta' OS=Roseiflexus castenholzii (strain DSM 13941 / HLO8) GN=rpoC PE=3 SV=1 | rca03020 RNA polymerase |
| A5USR7 | DNA-directed RNA polymerase subunit beta OS=Roseiflexus sp. (strain RS-1) GN=rpoB PE=3 SV=1 | rrs03020 RNA polymerase |
| A5USR6 | DNA-directed RNA polymerase subunit beta' OS=Roseiflexus sp. (strain RS-1) GN=rpoC PE=3 SV=1 | rrs03020 RNA polymerase |
| Q5LMQ5 | DNA-directed RNA polymerase subunit beta OS=Ruegeria pomeroyi (strain ATCC 700808 / DSM 15171 / DSS-3) GN=rpoB PE=3 SV=1 | sil03020 RNA polymerase |
| Q5LMQ6 | DNA-directed RNA polymerase subunit beta' OS=Ruegeria pomeroyi (strain ATCC 700808 / DSM 15171 / DSS-3) GN=rpoC PE=3 SV=1 | sil03020 RNA polymerase |
| Q1GK49 | DNA-directed RNA polymerase subunit beta OS=Ruegeria sp. (strain TM1040) GN=rpoB PE=3 SV=1 | sit03020 RNA polymerase |
| Q1GK48 | DNA-directed RNA polymerase subunit beta' OS=Ruegeria sp. (strain TM1040) GN=rpoC PE=3 SV=1 | sit03020 RNA polymerase |
| A1AX75 | DNA-directed RNA polymerase subunit beta OS=Ruthia magnifica subsp. Calyptogena magnifica GN=rpoB PE=3 SV=1 | rma03020 RNA polymerase |
| Q21M92 | DNA-directed RNA polymerase subunit beta' OS=Saccharophagus degradans (strain 2-40 / ATCC 43961 / DSM 17024) GN=rpoC PE=3 SV=1 | sde03020 RNA polymerase |
| Q2S1Q6 | DNA-directed RNA polymerase subunit beta' OS=Salinibacter ruber (strain DSM 13855 / M31) GN=rpoC PE=3 SV=1 | sru03020 RNA polymerase |
| A8M538 | DNA-directed RNA polymerase subunit beta' OS=Salinispora arenicola (strain CNS-205) GN=rpoC PE=3 SV=1 | saq03020 RNA polymerase |
| A1S211 | DNA-directed RNA polymerase subunit beta OS=Shewanella amazonensis (strain ATCC BAA-1098 / SB2B) GN=rpoB PE=3 SV=1 | saz03020 RNA polymerase |
| A1S212 | DNA-directed RNA polymerase subunit beta' OS=Shewanella amazonensis (strain ATCC BAA-1098 / SB2B) GN=rpoC PE=3 SV=1 | saz03020 RNA polymerase |
| A8G1F5 | DNA-directed RNA polymerase subunit beta OS=Shewanella sediminis (strain HAW-EB3) GN=rpoB PE=3 SV=1 | sse03020 RNA polymerase |
| Q9KW14 | DNA-directed RNA polymerase subunit beta OS=Shewanella violacea GN=rpoB PE=3 SV=1 | RNA polymerase |
| B1KMZ0 | DNA-directed RNA polymerase subunit beta OS=Shewanella woodyi (strain ATCC 51908 / MS32) GN=rpoB PE=3 SV=1 | swd03020 RNA polymerase |
| Q01VB2 | DNA-directed RNA polymerase subunit beta' OS=Solibacter usitatus (strain Ellin6076) GN=rpoC PE=3 SV=1 | sus03020 RNA polymerase |
| A9GRB4 | DNA-directed RNA polymerase subunit beta' OS=Sorangium cellulosum (strain So ce56) GN=rpoC PE=3 SV=1 | scl03020 RNA polymerase |
| A5VBZ8 | DNA-directed RNA polymerase subunit beta OS=Sphingomonas wittichii (strain RW1 / DSM 6014 / JCM 10273) GN=rpoB PE=3 SV=1 | swi03020 RNA polymerase |
| A5VBZ7 | DNA-directed RNA polymerase subunit beta' OS=Sphingomonas wittichii (strain RW1 / DSM 6014 / JCM 10273) GN=rpoC PE=3 SV=1 | swi03020 RNA polymerase |
| Q1GT24 | DNA-directed RNA polymerase subunit beta' OS=Sphingopyxis alaskensis (strain DSM 13593 / LMG 18877 / RB2256) GN=rpoC PE=3 SV=1 | sal03020 RNA polymerase |
| Q4L3K4 | DNA-directed RNA polymerase subunit beta' OS=Staphylococcus haemolyticus (strain JCSC1435) GN=rpoC PE=3 SV=1 | sha03020 RNA polymerase |
| Q8DS46 | DNA-directed RNA polymerase subunit beta OS=Streptococcus mutans serotype c (strain ATCC 700610 / UA159) GN=rpoB PE=3 SV=1 | smu03020 RNA polymerase |
| Q97NQ7 | DNA-directed RNA polymerase subunit beta OS=Streptococcus pneumoniae serotype 4 (strain ATCC BAA-334 / TIGR4) GN=rpoB PE=3 SV=1 | spn03020 RNA polymerase |
| Q5LXV3 | DNA-directed RNA polymerase subunit beta OS=Streptococcus thermophilus (strain CNRZ 1066) GN=rpoB PE=3 SV=1 | stc03020 RNA polymerase |
| Q82DQ5 | DNA-directed RNA polymerase subunit beta OS=Streptomyces avermitilis (strain ATCC 31267 / DSM 46492 / JCM 5070 / NBRC 14893 / NCIMB 12804 / NRRL 8165 / MA-4680) GN=rpoB PE=3 SV=2 | sma03020 RNA polymerase |
| Q82DQ4 | DNA-directed RNA polymerase subunit beta' OS=Streptomyces avermitilis (strain ATCC 31267 / DSM 46492 / JCM 5070 / NBRC 14893 / NCIMB 12804 / NRRL 8165 / MA-4680) GN=rpoC PE=3 SV=1 | sma03020 RNA polymerase |
| B1W441 | DNA-directed RNA polymerase subunit beta' OS=Streptomyces griseus subsp. griseus (strain JCM 4626 / NBRC 13350) GN=rpoC PE=3 SV=1 | sgr03020 RNA polymerase |
| A6Q6I2 | DNA-directed RNA polymerase subunit beta OS=Sulfurovum sp. (strain NBC37-1) GN=rpoB PE=3 SV=1 | sun03020 RNA polymerase |
| A6Q6I3 | DNA-directed RNA polymerase subunit beta' OS=Sulfurovum sp. (strain NBC37-1) GN=rpoC PE=3 SV=1 | sun03020 RNA polymerase |
| Q67JT3 | DNA-directed RNA polymerase subunit beta OS=Symbiobacterium thermophilum (strain T / IAM 14863) GN=rpoB PE=3 SV=1 | sth Symbiobacterium thermophilum |
| Q67JT4 | DNA-directed RNA polymerase subunit beta' OS=Symbiobacterium thermophilum (strain T / IAM 14863) GN=rpoC PE=3 SV=1 | sth Symbiobacterium thermophilum |
| A0LII4 | DNA-directed RNA polymerase subunit beta OS=Syntrophobacter fumaroxidans (strain DSM 10017 / MPOB) GN=rpoB PE=3 SV=1 | sfu03020 RNA polymerase |
| A0LII5 | DNA-directed RNA polymerase subunit beta' OS=Syntrophobacter fumaroxidans (strain DSM 10017 / MPOB) GN=rpoC PE=3 SV=1 | sfu03020 RNA polymerase |
| Q0AUH2 | DNA-directed RNA polymerase subunit beta OS=Syntrophomonas wolfei subsp. wolfei (strain DSM 2245B / Goettingen) GN=rpoB PE=3 SV=1 | swo03020 RNA polymerase |
| Q2LQ87 | DNA-directed RNA polymerase subunit beta OS=Syntrophus aciditrophicus (strain SB) GN=rpoB PE=3 SV=1 | sat03020 RNA polymerase |
| Q2LQ86 | DNA-directed RNA polymerase subunit beta' OS=Syntrophus aciditrophicus (strain SB) GN=rpoC PE=3 SV=1 | sat03020 RNA polymerase |
| B0K5G9 | DNA-directed RNA polymerase subunit beta' OS=Thermoanaerobacter sp. (strain X514) GN=rpoC PE=3 SV=2 | tex03020 RNA polymerase |
| Q47LI5 | DNA-directed RNA polymerase subunit beta OS=Thermobifida fusca (strain YX) GN=rpoB PE=3 SV=1 | tfu03020 RNA polymerase |
| B5YFV7 | DNA-directed RNA polymerase subunit beta' OS=Thermodesulfovibrio yellowstonii (strain ATCC 51303 / DSM 11347 / YP87) GN=rpoC PE=3 SV=1 | tye03020 RNA polymerase |
| A6LKB8 | DNA-directed RNA polymerase subunit beta OS=Thermosipho melanesiensis (strain DSM 12029 / CIP 104789 / BI429) GN=rpoB PE=3 SV=1 | tme03020 RNA polymerase |
| B9KBJ4 | DNA-directed RNA polymerase subunit beta' OS=Thermotoga neapolitana (strain ATCC 49049 / DSM 4359 / NS-E) GN=rpoC PE=3 SV=1 | tna03020 RNA polymerase |
| A5IJW3 | DNA-directed RNA polymerase subunit beta OS=Thermotoga petrophila (strain RKU-1 / ATCC BAA-488 / DSM 13995) GN=rpoB PE=3 SV=1 | tpt03020 RNA polymerase |
| A5IJW2 | DNA-directed RNA polymerase subunit beta' OS=Thermotoga petrophila (strain RKU-1 / ATCC BAA-488 / DSM 13995) GN=rpoC PE=3 SV=1 | tpt03020 RNA polymerase |
| B1L934 | DNA-directed RNA polymerase subunit beta' OS=Thermotoga sp. (strain RQ2) GN=rpoC PE=3 SV=1 | trq03020 RNA polymerase |
| Q8RQE9 | DNA-directed RNA polymerase subunit beta OS=Thermus thermophilus (strain HB8 / ATCC 27634 / DSM 579) GN=rpoB PE=1 SV=1 | ttj03020 RNA polymerase |
| Q3SLQ5 | DNA-directed RNA polymerase subunit beta' OS=Thiobacillus denitrificans (strain ATCC 25259) GN=rpoC PE=3 SV=1 | tbd03020 RNA polymerase |
| Q31IY9 | DNA-directed RNA polymerase subunit beta OS=Thiomicrospira crunogena (strain XCL-2) GN=rpoB PE=3 SV=1 | tcx03020 RNA polymerase |
| C4LBV1 | DNA-directed RNA polymerase subunit beta' OS=Tolumonas auensis (strain DSM 9187 / TA4) GN=rpoC PE=3 SV=1 | tau03020 RNA polymerase |
| Q73JJ7 | DNA-directed RNA polymerase subunit beta OS=Treponema denticola (strain ATCC 35405 / CIP 103919 / DSM 14222) GN=rpoB PE=3 SV=1 | tde03020 RNA polymerase |
| Q73JJ8 | DNA-directed RNA polymerase subunit beta' OS=Treponema denticola (strain ATCC 35405 / CIP 103919 / DSM 14222) GN=rpoC PE=3 SV=1 | tde03020 RNA polymerase |
| O83270 | DNA-directed RNA polymerase subunit beta' OS=Treponema pallidum (strain Nichols) GN=rpoC PE=3 SV=1 | tpa03020 RNA polymerase |
| Q820D6 | DNA-directed RNA polymerase subunit beta' OS=Tropheryma whipplei (strain Twist) GN=rpoC PE=3 SV=1 | twh03020 RNA polymerase |
| B1GZ76 | DNA-directed RNA polymerase subunit beta' OS=Uncultured termite group 1 bacterium phylotype Rs-D17 GN=rpoC PE=3 SV=1 | rsd03020 RNA polymerase |
| Q9PQV6 | DNA-directed RNA polymerase subunit beta OS=Ureaplasma parvum serovar 3 (strain ATCC 700970) GN=rpoB PE=3 SV=1 | uur03020 RNA polymerase |
| A1WK55 | DNA-directed RNA polymerase subunit beta OS=Verminephrobacter eiseniae (strain EF01-2) GN=rpoB PE=3 SV=1 | vei03020 RNA polymerase |
| A1WK56 | DNA-directed RNA polymerase subunit beta' OS=Verminephrobacter eiseniae (strain EF01-2) GN=rpoC PE=3 SV=1 | vei03020 RNA polymerase |
| A5CW24 | DNA-directed RNA polymerase subunit beta' OS=Vesicomyosocius okutanii subsp. Calyptogena okutanii (strain HA) GN=rpoC PE=3 SV=1 | vok03020 RNA polymerase |
| Q9KV29 | DNA-directed RNA polymerase subunit beta' OS=Vibrio cholerae serotype O1 (strain ATCC 39315 / El Tor Inaba N16961) GN=rpoC PE=3 SV=1 | vch03020 RNA polymerase |
| Q5E239 | DNA-directed RNA polymerase subunit beta' OS=Vibrio fischeri (strain ATCC 700601 / ES114) GN=rpoC PE=3 SV=2 | vfi03020 RNA polymerase |
| Q8DD20 | DNA-directed RNA polymerase subunit beta OS=Vibrio vulnificus (strain CMCP6) GN=rpoB PE=3 SV=1 | vvu03020 RNA polymerase |
| Q8DD19 | DNA-directed RNA polymerase subunit beta' OS=Vibrio vulnificus (strain CMCP6) GN=rpoC PE=3 SV=1 | vvu03020 RNA polymerase |
| Q8D233 | DNA-directed RNA polymerase subunit beta OS=Wigglesworthia glossinidia brevipalpis GN=rpoB PE=3 SV=1 | wbr03020 RNA polymerase |
| Q8D232 | DNA-directed RNA polymerase subunit beta' OS=Wigglesworthia glossinidia brevipalpis GN=rpoC PE=3 SV=1 | wbr03020 RNA polymerase |
| A7IKQ1 | DNA-directed RNA polymerase subunit beta' OS=Xanthobacter autotrophicus (strain ATCC BAA-1158 / Py2) GN=rpoC PE=3 SV=1 | xau03020 RNA polymerase |
| Q4URD2 | DNA-directed RNA polymerase subunit beta OS=Xanthomonas campestris pv. campestris (strain 8004) GN=rpoB PE=3 SV=1 | xcb03020 RNA polymerase |
| Q3BWZ0 | DNA-directed RNA polymerase subunit beta' OS=Xanthomonas campestris pv. vesicatoria (strain 85-10) GN=rpoC PE=3 SV=1 | xcv03020 RNA polymerase |
| Q5GWS6 | DNA-directed RNA polymerase subunit beta OS=Xanthomonas oryzae pv. oryzae (strain KACC10331 / KXO85) GN=rpoB PE=3 SV=1 | xoo03020 RNA polymerase |
| Q9PA87 | DNA-directed RNA polymerase subunit beta' OS=Xylella fastidiosa (strain 9a5c) GN=rpoC PE=3 SV=2 | xfa03020 RNA polymerase |
| A1JII0 | DNA-directed RNA polymerase subunit beta OS=Yersinia enterocolitica serotype O:8 / biotype 1B (strain NCTC 13174 / 8081) GN=rpoB PE=3 SV=1 | yen03020 RNA polymerase |
| Q5NPK4 | DNA-directed RNA polymerase subunit beta' OS=Zymomonas mobilis subsp. mobilis (strain ATCC 31821 / ZM4 / CP4) GN=rpoC PE=3 SV=2 | zmo03020 RNA polymerase |
| A9VHU6 | Elongation factor 4 OS=Bacillus weihenstephanensis (strain KBAB4) GN=lepA PE=3 SV=1 | GTP-binding protein LepA |
| O51115 | Elongation factor 4 OS=Borrelia burgdorferi (strain ATCC 35210 / B31 / CIP 102532 / DSM 4680) GN=lepA PE=3 SV=2 | elongation factor EF-4 |
| Q3B2V1 | Elongation factor 4 OS=Chlorobium luteolum (strain DSM 273 / 2530) GN=lepA PE=3 SV=1 | GTP-binding protein LepA |
| Q97JJ6 | Elongation factor 4 OS=Clostridium acetobutylicum (strain ATCC 824 / DSM 792 / JCM 1419 / LMG 5710 / VKM B-1787) GN=lepA PE=3 SV=1 | GTP-binding protein LepA |
| A9KKU4 | Elongation factor 4 OS=Clostridium phytofermentans (strain ATCC 700394 / DSM 18823 / ISDg) GN=lepA PE=3 SV=1 | GTP-binding protein LepA |
| A4J7F8 | Elongation factor 4 OS=Desulfotomaculum reducens (strain MI-1) GN=lepA PE=3 SV=1 | GTP-binding protein LepA |
| C4Z541 | Elongation factor 4 OS=Eubacterium eligens (strain ATCC 27750 / VPI C15-48) GN=lepA PE=3 SV=1 | GTP-binding protein LepA |
| B1XZN0 | Elongation factor 4 OS=Leptothrix cholodnii (strain ATCC 51168 / LMG 8142 / SP-6) GN=lepA PE=3 SV=1 | GTP-binding protein LepA |
| A2SDH0 | Elongation factor 4 OS=Methylibium petroleiphilum (strain ATCC BAA-1232 / LMG 22953 / PM1) GN=lepA PE=3 SV=1 | GTP-binding protein LepA |
| Q1D6M1 | Elongation factor 4 OS=Myxococcus xanthus (strain DK 1622) GN=lepA PE=3 SV=1 | GTP-binding protein LepA |
| B2J0M4 | Elongation factor 4 OS=Nostoc punctiforme (strain ATCC 29133 / PCC 73102) GN=lepA PE=3 SV=1 | GTP-binding protein LepA |
| A2BPP8 | Elongation factor 4 OS=Prochlorococcus marinus (strain AS9601) GN=lepA PE=3 SV=1 | GTP-binding protein LepA |
| A2CBG9 | Elongation factor 4 OS=Prochlorococcus marinus (strain MIT 9303) GN=lepA PE=3 SV=1 | Membrane GTPase LepA |
| Q7VDF7 | Elongation factor 4 OS=Prochlorococcus marinus (strain SARG / CCMP1375 / SS120) GN=lepA PE=3 SV=1 | elongation factor LepA |
| C1AUN7 | Elongation factor 4 OS=Rhodococcus opacus (strain B4) GN=lepA PE=3 SV=1 | elongation factor LepA |
| Q01SV7 | Elongation factor 4 OS=Solibacter usitatus (strain Ellin6076) GN=lepA PE=3 SV=1 | GTP-binding protein LepA |
| B9DNK4 | Elongation factor 4 OS=Staphylococcus carnosus (strain TM300) GN=lepA PE=3 SV=1 | GTP-binding protein LepA |
| Q31R08 | Elongation factor 4 OS=Synechococcus elongatus (strain PCC 7942) GN=lepA PE=3 SV=1 | GTP-binding protein LepA |
| A5GRE6 | Elongation factor 4 OS=Synechococcus sp. (strain RCC307) GN=lepA PE=3 SV=1 | GTP-binding protein LepA |
| Q3BVV9 | Elongation factor 4 OS=Xanthomonas campestris pv. vesicatoria (strain 85-10) GN=lepA PE=3 SV=1 | GTP-binding protein LepA |
| Q9PBA1 | Elongation factor 4 OS=Xylella fastidiosa (strain 9a5c) GN=lepA PE=3 SV=1 | GTP-binding protein LepA |
| C5CC67 | Elongation factor G OS=Micrococcus luteus (strain ATCC 4698 / DSM 20030 / JCM 1464 / NBRC 3333 / NCIMB 9278 / NCTC 2665 / VKM Ac-2230) GN=fusA PE=3 SV=1 | Translation elongation factor 2 (EF-2/EF-G) |
| A8YTJ4 | Endonuclease MutS2 OS=Lactobacillus helveticus (strain DPC 4571) GN=mutS2 PE=3 SV=1 | DNA mismatch repair protein |
| Q11LY8 | Exodeoxyribonuclease 7 large subunit OS=Chelativorans sp. (strain BNC1) GN=xseA PE=3 SV=1 | [mes03430](https://www.genome.jp/kegg-bin/show_pathway?mes03430+Meso_0182)  Mismatch repair |
| Q7N0C2 | Glutamate-ammonia-ligase adenylyltransferase OS=Photorhabdus luminescens subsp. laumondii (strain DSM 15139 / CIP 105565 / TT01) GN=glnE PE=3 SV=1 | glutamate-ammonia-ligase adenylyltransferase (glutamine-synthetase adenylyltransferase) (ATASE) |
| Q985F6 | Glutamate-ammonia-ligase adenylyltransferase OS=Rhizobium loti (strain MAFF303099) GN=glnE PE=3 SV=1 | glutamate-ammonia-ligase; adenylyltransferase |
| A1S909 | Glutamate-ammonia-ligase adenylyltransferase OS=Shewanella amazonensis (strain ATCC BAA-1098 / SB2B) GN=glnE PE=3 SV=1 | (Glutamate-ammonia-ligase) adenylyltransferase) |
| Q8PPY3 | Glutamate-ammonia-ligase adenylyltransferase OS=Xanthomonas axonopodis pv. citri (strain 306) GN=glnE PE=3 SV=1 | glutamine synthetase adenylyltransferase |
| O86781 | Glutamine--fructose-6-phosphate aminotransferase [isomerizing] OS=Streptomyces coelicolor (strain ATCC BAA-471 / A3(2) / M145) GN=glmS PE=3 SV=3 | glucosamine--fructose-6-phosphate aminotransferase |
| Q89KR6 | Glutamine--tRNA ligase OS=Bradyrhizobium diazoefficiens (strain JCM 10833 / IAM 13628 / NBRC 14792 / USDA 110) GN=glnS PE=3 SV=1 | Aminoacyl-tRNA biosynthesis  Metabolic pathways |
| B8D7U5 | Glutamine--tRNA ligase OS=Buchnera aphidicola subsp. Acyrthosiphon pisum (strain Tuc7) GN=glnS PE=3 SV=1 | Aminoacyl-tRNA biosynthesis  Metabolic pathways |
| Q8K9E1 | Glutamine--tRNA ligase OS=Buchnera aphidicola subsp. Schizaphis graminum (strain Sg) GN=glnS PE=3 SV=2 | Aminoacyl-tRNA biosynthesis  Metabolic pathways |
| C5BGA4 | Glutamine--tRNA ligase OS=Edwardsiella ictaluri (strain 93-146) GN=glnS PE=3 SV=1 | Aminoacyl-tRNA biosynthesis  Metabolic pathways |
| B7L9L7 | Glutamine--tRNA ligase OS=Escherichia coli (strain 55989 / EAEC) GN=glnS PE=3 SV=1 | Aminoacyl-tRNA biosynthesis  Metabolic pathways |
| Q6D7J7 | Glutamine--tRNA ligase OS=Pectobacterium atrosepticum (strain SCRI 1043 / ATCC BAA-672) GN=glnS PE=3 SV=1 | Aminoacyl-tRNA biosynthesis  Metabolic pathways |
| Q188C8 | Glutamine--tRNA ligase OS=Peptoclostridium difficile (strain 630) GN=glnS PE=3 SV=1 | Aminoacyl-tRNA biosynthesis  Metabolic pathways |
| Q7N743 | Glutamine--tRNA ligase OS=Photorhabdus luminescens subsp. laumondii (strain DSM 15139 / CIP 105565 / TT01) GN=glnS PE=3 SV=1 | Aminoacyl-tRNA biosynthesis  Metabolic pathways |
| A1SS83 | Glutamine--tRNA ligase OS=Psychromonas ingrahamii (strain 37) GN=glnS PE=3 SV=1 | Aminoacyl-tRNA biosynthesis  Metabolic pathways |
| B2U846 | Glutamine--tRNA ligase OS=Ralstonia pickettii (strain 12J) GN=glnS PE=3 SV=1 | Aminoacyl-tRNA biosynthesis  Metabolic pathways |
| A7MT45 | Glutamine--tRNA ligase OS=Vibrio campbellii (strain ATCC BAA-1116 / BB120) GN=glnS PE=3 SV=1 | Aminoacyl-tRNA biosynthesis  Metabolic pathways |
| Q88ZF1 | Glycerol kinase 1 OS=Lactobacillus plantarum (strain ATCC BAA-793 / NCIMB 8826 / WCFS1) GN=glpK1 PE=3 SV=1 | Aminoacyl-tRNA biosynthesis  Metabolic pathways |
| A1TZQ4 | GTPase Der OS=Marinobacter hydrocarbonoclasticus (strain ATCC 700491 / DSM 11845 / VT8) GN=der PE=3 SV=1 | Aminoacyl-tRNA biosynthesis  Metabolic pathways |
| A7NFV2 | Histidinol-phosphate aminotransferase OS=Roseiflexus castenholzii (strain DSM 13941 / HLO8) GN=hisC PE=3 SV=1 | Histidine metabolism  Tyrosine metabolism  Phenylalanine metabolism  Phenylalanine, tyrosine and tryptophan biosynthesis  Novobiocin biosynthesis  Metabolic pathways  Biosynthesis of secondary metabolites  Biosynthesis of antibiotics  Biosynthesis of amino acids |
| P27951 | IgA FC receptor OS=Streptococcus agalactiae GN=bag PE=1 SV=1 | Receptor protein |
| P02976 | Immunoglobulin G-binding protein A OS=Staphylococcus aureus (strain NCTC 8325) GN=spa PE=1 SV=3 | sao05150  *Staphylococcus aureus* infection |
| P29166 | Iron hydrogenase 1 OS=Clostridium pasteurianum PE=1 SV=1 |  |
| A6Q4I1 | Isoleucine--tRNA ligase OS=Nitratiruptor sp. (strain SB155-2) GN=ileS PE=3 SV=1 | nis00970 Aminoacyl-tRNA biosynthesis |
| Q7TV01 | Leucine--tRNA ligase OS=Prochlorococcus marinus (strain MIT 9313) GN=leuS PE=3 SV=1 | pmt Prochlorococcus marinus MIT 9313 |
| A9F881 | LexA repressor OS=Sorangium cellulosum (strain So ce56) GN=lexA PE=3 SV=1 | [scl](https://www.genome.jp/kegg-bin/show_organism?org=scl)  Sorangium cellulosum So ce56  LexA repressor |
| Q28W35 | Light-independent protochlorophyllide reductase subunit B OS=Jannaschia sp. (strain CCS1) GN=bchB PE=3 SV=1 | Porphyrin and chlorophyll metabolism  Metabolic pathways  Biosynthesis of secondary metabolites |
| Q16DS0 | Light-independent protochlorophyllide reductase subunit B OS=Roseobacter denitrificans (strain ATCC 33942 / OCh 114) GN=bchB PE=3 SV=1 | Porphyrin and chlorophyll metabolism  Metabolic pathways  Biosynthesis of secondary metabolites |
| Q4FTQ5 | Lipoyl synthase OS=Psychrobacter arcticus (strain DSM 17307 / 273-4) GN=lipA PE=3 SV=1 | Lipoic acid metabolism  Metabolic pathways |
| B0S2N4 | Lon protease OS=Finegoldia magna (strain ATCC 29328) GN=lon PE=3 SV=1 | ATP-dependent protease |
| Q9ZD92 | Lon protease OS=Rickettsia prowazekii (strain Madrid E) GN=lon PE=3 SV=1 | ATP-dependent protease LA (lon) |
| P10547 | Lysostaphin OS=Staphylococcus simulans GN=lss PE=1 SV=2 | lysostaphin |
| P14565 | Multifunctional conjugation protein TraI OS=Escherichia coli (strain K12) GN=traI PE=1 SV=2 | lysostaphin |
| A8LVS8 | Na(+)/H(+) antiporter NhaA 1 OS=Salinispora arenicola (strain CNS-205) GN=nhaA1 PE=3 SV=1 | Na+/H+ antiporter NhaA |
| Q06457 | Nitrate reductase OS=Klebsiella oxytoca GN=nasA PE=2 SV=2 | outer membrane usher protein |
| P33341 | Outer membrane usher protein YehB OS=Escherichia coli (strain K12) GN=yehB PE=2 SV=1 | outer membrane usher protein |
| O67886 | Oxygen-independent coproporphyrinogen-III oxidase OS=Aquifex aeolicus (strain VF5) GN=hemN PE=3 SV=1 | Na+/H+ antiporter NhaA |
| Q6F823 | Peptide chain release factor 3 OS=Acinetobacter baylyi (strain ATCC 33305 / BD413 / ADP1) GN=prfC PE=3 SV=1 | oxygen-independent coproporphyrinogen III oxidase |
| Q5P409 | Peptide chain release factor 3 OS=Aromatoleum aromaticum (strain EbN1) GN=prfC PE=3 SV=1 | peptide chain release factor 3 |
| A8ALY7 | Peptide chain release factor 3 OS=Citrobacter koseri (strain ATCC BAA-895 / CDC 4225-83 / SGSC4696) GN=prfC PE=3 SV=1 | peptide chain release factor 3 |
| Q1H0I2 | Peptide chain release factor 3 OS=Methylobacillus flagellatus (strain KT / ATCC 51484 / DSM 6875) GN=prfC PE=3 SV=1 | peptide chain release factor 3 |
| A6V0K2 | Peptide chain release factor 3 OS=Pseudomonas aeruginosa (strain PA7) GN=prfC PE=3 SV=1 | peptide chain release factor 3 |
| Q31H08 | Peptide chain release factor 3 OS=Thiomicrospira crunogena (strain XCL-2) GN=prfC PE=3 SV=1 | regulation of translational termination |
| O07532 | Peptidoglycan endopeptidase LytF OS=Bacillus subtilis (strain 168) GN=lytF PE=1 SV=2 | eptidoglycan DL-endopeptidase LytF |
| A6VQY7 | Periplasmic nitrate reductase OS=Actinobacillus succinogenes (strain ATCC 55618 / 130Z) GN=napA PE=3 SV=1 | periplasmic nitrate reductase NapA |
| A9I7M5 | Periplasmic nitrate reductase OS=Bordetella petrii (strain ATCC BAA-461 / DSM 12804 / CCUG 43448) GN=napA PE=3 SV=1 | periplasmic nitrate reductase NapA |
| Q47A87 | Periplasmic nitrate reductase OS=Dechloromonas aromatica (strain RCB) GN=napA PE=3 SV=1 | periplasmic nitrate reductase NapA |
| A5UAE1 | Periplasmic nitrate reductase OS=Haemophilus influenzae (strain PittEE) GN=napA PE=3 SV=1 | periplasmic nitrate reductase NapA |
| B1Y6A6 | Periplasmic nitrate reductase OS=Leptothrix cholodnii (strain ATCC 51168 / LMG 8142 / SP-6) GN=napA PE=3 SV=1 | periplasmic nitrate reductase NapA |
| B0UCB6 | Periplasmic nitrate reductase OS=Methylobacterium sp. (strain 4-46) GN=napA PE=3 SV=1 | periplasmic nitrate reductase NapA |
| Q9CKL8 | Periplasmic nitrate reductase OS=Pasteurella multocida (strain Pm70) GN=napA PE=3 SV=1 | periplasmic nitrate reductase NapA |
| Q7M962 | Periplasmic nitrate reductase OS=Wolinella succinogenes (strain ATCC 29543 / DSM 1740 / LMG 7466 / NCTC 11488 / FDC 602W) GN=napA PE=3 SV=2 | periplasmic nitrate reductase NapA |
| Q5F588 | Phosphomethylpyrimidine synthase OS=Neisseria gonorrhoeae (strain ATCC 700825 / FA 1090) GN=thiC PE=3 SV=1 | phosphomethylpyrimidine synthase |
| Q2SK05 | Phosphoribosylformylglycinamidine synthase OS=Hahella chejuensis (strain KCTC 2396) GN=purL PE=3 SV=1 | phosphoribosylformylglycinamidine synthase |
| Q5QWY0 | Phosphoribosylformylglycinamidine synthase OS=Idiomarina loihiensis (strain ATCC BAA-735 / DSM 15497 / L2-TR) GN=purL PE=3 SV=1 | phosphoribosylformylglycinamidine synthase |
| Q6D238 | Phosphoribosylformylglycinamidine synthase OS=Pectobacterium atrosepticum (strain SCRI 1043 / ATCC BAA-672) GN=purL PE=3 SV=1 | phosphoribosylformylglycinamidine synthase |
| Q6LU24 | Phosphoribosylformylglycinamidine synthase OS=Photobacterium profundum (strain SS9) GN=purL PE=3 SV=1 | phosphoribosylformylglycinamidine synthase |
| Q15R69 | Phosphoribosylformylglycinamidine synthase OS=Pseudoalteromonas atlantica (strain T6c / ATCC BAA-1087) GN=purL PE=3 SV=1 | phosphoribosylformylglycinamidine synthase |
| Q3KHL4 | Phosphoribosylformylglycinamidine synthase OS=Pseudomonas fluorescens (strain Pf0-1) GN=purL PE=3 SV=1 | phosphoribosylformylglycinamidine synthase |
| Q886W6 | Phosphoribosylformylglycinamidine synthase OS=Pseudomonas syringae pv. tomato (strain DC3000) GN=purL PE=3 SV=1 | phosphoribosylformylglycinamidine synthase |
| Q085S1 | Phosphoribosylformylglycinamidine synthase OS=Shewanella frigidimarina (strain NCIMB 400) GN=purL PE=3 SV=1 | phosphoribosylformylglycinamidine synthase |
| Q0HKU9 | Phosphoribosylformylglycinamidine synthase OS=Shewanella sp. (strain MR-4) GN=purL PE=3 SV=1 | phosphoribosylformylglycinamidine synthase |
| Q3YYZ8 | Phosphoribosylformylglycinamidine synthase OS=Shigella sonnei (strain Ss046) GN=purL PE=3 SV=3 | phosphoribosylformylglycinamidine synthase |
| Q9KTN2 | Phosphoribosylformylglycinamidine synthase OS=Vibrio cholerae serotype O1 (strain ATCC 39315 / El Tor Inaba N16961) GN=purL PE=3 SV=1 | phosphoribosylformylglycinamidine synthase |
| Q7MN70 | Phosphoribosylformylglycinamidine synthase OS=Vibrio vulnificus (strain YJ016) GN=purL PE=3 SV=2 | Purine metabolism  Metabolic pathways  Biosynthesis of secondary metabolites  Biosynthesis of antibiotics |
| Q7NFT5 | Photosystem I P700 chlorophyll a apoprotein A2 OS=Gloeobacter violaceus (strain PCC 7421) GN=psaB PE=1 SV=1 | photosystem I P700 chlorophyll a apoprotein A2 |
| Q73VJ1 | Polyphosphate kinase OS=Mycobacterium paratuberculosis (strain ATCC BAA-968 / K-10) GN=ppk PE=3 SV=1 | polyphosphate kinase |
| Q6FF12 | Polyribonucleotide nucleotidyltransferase OS=Acinetobacter baylyi (strain ATCC 33305 / BD413 / ADP1) GN=pnp PE=3 SV=1 | polyribonucleotide nucleotidyltransferase |
| Q0VSR7 | Polyribonucleotide nucleotidyltransferase OS=Alcanivorax borkumensis (strain ATCC 700651 / DSM 11573 / NCIMB 13689 / SK2) GN=pnp PE=3 SV=1 | polyribonucleotide nucleotidyltransferase |
| B6ENE6 | Polyribonucleotide nucleotidyltransferase OS=Aliivibrio salmonicida (strain LFI1238) GN=pnp PE=3 SV=2 | polyribonucleotide nucleotidyltransferase |
| Q0A7A1 | Polyribonucleotide nucleotidyltransferase OS=Alkalilimnicola ehrlichii (strain ATCC BAA-1101 / DSM 17681 / MLHE-1) GN=pnp PE=3 SV=2 | polyribonucleotide nucleotidyltransferase |
| A7H9F8 | Polyribonucleotide nucleotidyltransferase OS=Anaeromyxobacter sp. (strain Fw109-5) GN=pnp PE=3 SV=1 | polyribonucleotide nucleotidyltransferase |
| Q2GJV5 | Polyribonucleotide nucleotidyltransferase OS=Anaplasma phagocytophilum (strain HZ) GN=pnp PE=3 SV=1 | polyribonucleotide nucleotidyltransferase |
| A8IGA3 | Polyribonucleotide nucleotidyltransferase OS=Azorhizobium caulinodans (strain ATCC 43989 / DSM 5975 / JCM 20966 / NBRC 14845 / NCIMB 13405 / ORS 571) GN=pnp PE=3 SV=2 | polyribonucleotide nucleotidyltransferase |
| A0RHH8 | Polyribonucleotide nucleotidyltransferase OS=Bacillus thuringiensis (strain Al Hakam) GN=pnp PE=3 SV=1 | polyribonucleotide nucleotidyltransferase |
| A1UU54 | Polyribonucleotide nucleotidyltransferase OS=Bartonella bacilliformis (strain ATCC 35685 / KC583) GN=pnp PE=3 SV=1 | polyribonucleotide nucleotidyltransferase |
| A9IMR9 | Polyribonucleotide nucleotidyltransferase OS=Bartonella tribocorum (strain CIP 105476 / IBS 506) GN=pnp PE=3 SV=1 | polyribonucleotide nucleotidyltransferase |
| B2ICY4 | Polyribonucleotide nucleotidyltransferase OS=Beijerinckia indica subsp. indica (strain ATCC 9039 / DSM 1715 / NCIB 8712) GN=pnp PE=3 SV=1 | polyribonucleotide nucleotidyltransferase |
| C0ZF43 | Polyribonucleotide nucleotidyltransferase OS=Brevibacillus brevis (strain 47 / JCM 6285 / NBRC 100599) GN=pnp PE=3 SV=1 | polyribonucleotide nucleotidyltransferase |
| Q2YQR3 | Polyribonucleotide nucleotidyltransferase OS=Brucella abortus (strain 2308) GN=pnp PE=3 SV=1 | polyribonucleotide nucleotidyltransferase |
| B8GWZ0 | Polyribonucleotide nucleotidyltransferase OS=Caulobacter crescentus (strain NA1000 / CB15N) GN=pnp PE=3 SV=1 | polyribonucleotide nucleotidyltransferase |
| Q11BC3 | Polyribonucleotide nucleotidyltransferase OS=Chelativorans sp. (strain BNC1) GN=pnp PE=3 SV=1 | polyribonucleotide nucleotidyltransferase |
| Q5L5B4 | Polyribonucleotide nucleotidyltransferase OS=Chlamydia abortus (strain DSM 27085 / S26/3) GN=pnp PE=3 SV=1 | polyribonucleotide nucleotidyltransferase |
| Q9Z6R0 | Polyribonucleotide nucleotidyltransferase OS=Chlamydia pneumoniae GN=pnp PE=3 SV=1 | polyribonucleotide nucleotidyltransferase |
| Q822C1 | Polyribonucleotide nucleotidyltransferase OS=Chlamydophila caviae (strain GPIC) GN=pnp PE=3 SV=1 | polyribonucleotide nucleotidyltransferase |
| Q3APY4 | Polyribonucleotide nucleotidyltransferase OS=Chlorobium chlorochromatii (strain CaD3) GN=pnp PE=3 SV=1 | polyribonucleotide nucleotidyltransferase |
| B3QY14 | Polyribonucleotide nucleotidyltransferase OS=Chloroherpeton thalassium (strain ATCC 35110 / GB-78) GN=pnp PE=3 SV=1 | polyribonucleotide nucleotidyltransferase |
| A8LKE7 | Polyribonucleotide nucleotidyltransferase OS=Dinoroseobacter shibae (strain DSM 16493 / NCIMB 14021 / DFL 12) GN=pnp PE=3 SV=1 | polyribonucleotide nucleotidyltransferase |
| Q2GGA4 | Polyribonucleotide nucleotidyltransferase OS=Ehrlichia chaffeensis (strain ATCC CRL-10679 / Arkansas) GN=pnp PE=3 SV=1 | polyribonucleotide nucleotidyltransferase |
| B5EI63 | Polyribonucleotide nucleotidyltransferase OS=Geobacter bemidjiensis (strain Bem / ATCC BAA-1014 / DSM 16622) GN=pnp PE=3 SV=1 | polyribonucleotide nucleotidyltransferase |
| A5GF91 | Polyribonucleotide nucleotidyltransferase OS=Geobacter uraniireducens (strain Rf4) GN=pnp PE=3 SV=1 | polyribonucleotide nucleotidyltransferase |
| A9HF35 | Polyribonucleotide nucleotidyltransferase OS=Gluconacetobacter diazotrophicus (strain ATCC 49037 / DSM 5601 / PAl5) GN=pnp PE=3 SV=1 | polyribonucleotide nucleotidyltransferase |
| Q2SMK9 | Polyribonucleotide nucleotidyltransferase OS=Hahella chejuensis (strain KCTC 2396) GN=pnp PE=3 SV=1 | polyribonucleotide nucleotidyltransferase |
| A1WXU7 | Polyribonucleotide nucleotidyltransferase OS=Halorhodospira halophila (strain DSM 244 / SL1) GN=pnp PE=3 SV=1 | polyribonucleotide nucleotidyltransferase |
| Q0AK62 | Polyribonucleotide nucleotidyltransferase OS=Maricaulis maris (strain MCS10) GN=pnp PE=3 SV=1 | polyribonucleotide nucleotidyltransferase |
| B8IGX3 | Polyribonucleotide nucleotidyltransferase OS=Methylobacterium nodulans (strain LMG 21967 / CNCM I-2342 / ORS 2060) GN=pnp PE=3 SV=1 | polyribonucleotide nucleotidyltransferase |
| B8EP09 | Polyribonucleotide nucleotidyltransferase OS=Methylocella silvestris (strain DSM 15510 / CIP 108128 / LMG 27833 / NCIMB 13906 / BL2) GN=pnp PE=3 SV=1 | polyribonucleotide nucleotidyltransferase |
| B6JCR8 | Polyribonucleotide nucleotidyltransferase OS=Oligotropha carboxidovorans (strain ATCC 49405 / DSM 1227 / KCTC 32145 / OM5) GN=pnp PE=3 SV=1 | polyribonucleotide nucleotidyltransferase |
| Q6YQX2 | Polyribonucleotide nucleotidyltransferase OS=Onion yellows phytoplasma (strain OY-M) GN=pnp PE=3 SV=1 | polyribonucleotide nucleotidyltransferase |
| A1B5P9 | Polyribonucleotide nucleotidyltransferase OS=Paracoccus denitrificans (strain Pd 1222) GN=pnp PE=3 SV=2 | polyribonucleotide nucleotidyltransferase |
| Q6LUI8 | Polyribonucleotide nucleotidyltransferase OS=Photobacterium profundum (strain SS9) GN=pnp PE=3 SV=1 | polyribonucleotide nucleotidyltransferase |
| Q6MDI2 | Polyribonucleotide nucleotidyltransferase OS=Protochlamydia amoebophila (strain UWE25) GN=pnp PE=3 SV=2 | polyribonucleotide nucleotidyltransferase |
| B0KHX3 | Polyribonucleotide nucleotidyltransferase OS=Pseudomonas putida (strain GB-1) GN=pnp PE=3 SV=1 | polyribonucleotide nucleotidyltransferase |
| Q1MN44 | Polyribonucleotide nucleotidyltransferase OS=Rhizobium leguminosarum bv. viciae (strain 3841) GN=pnp PE=3 SV=1 | polyribonucleotide nucleotidyltransferase |
| Q2RMR6 | Polyribonucleotide nucleotidyltransferase OS=Rhodospirillum rubrum (strain ATCC 11170 / ATH 1.1.1 / DSM 467 / LMG 4362 / NCIB 8255 / S1) GN=pnp PE=3 SV=1 | polyribonucleotide nucleotidyltransferase |
| Q5LN23 | Polyribonucleotide nucleotidyltransferase OS=Ruegeria pomeroyi (strain ATCC 700808 / DSM 15171 / DSS-3) GN=pnp PE=3 SV=1 | polyribonucleotide nucleotidyltransferase |
| Q1GVQ8 | Polyribonucleotide nucleotidyltransferase OS=Sphingopyxis alaskensis (strain DSM 13593 / LMG 18877 / RB2256) GN=pnp PE=3 SV=1 | polyribonucleotide nucleotidyltransferase |
| Q2LWT4 | Polyribonucleotide nucleotidyltransferase OS=Syntrophus aciditrophicus (strain SB) GN=pnp PE=3 SV=1 | polyribonucleotide nucleotidyltransferase |
| Q31GJ9 | Polyribonucleotide nucleotidyltransferase OS=Thiomicrospira crunogena (strain XCL-2) GN=pnp PE=3 SV=1 | polyribonucleotide nucleotidyltransferase |
| A7MZI2 | Polyribonucleotide nucleotidyltransferase OS=Vibrio campbellii (strain ATCC BAA-1116 / BB120) GN=pnp PE=3 SV=1 | polyribonucleotide nucleotidyltransferase |
| Q5GTR3 | Polyribonucleotide nucleotidyltransferase OS=Wolbachia sp. subsp. Brugia malayi (strain TRS) GN=pnp PE=3 SV=1 | polyribonucleotide nucleotidyltransferase |
| P0A523 | Probable ATP-dependent Clp protease ATP-binding subunit OS=Mycobacterium bovis (strain ATCC BAA-935 / AF2122/97) GN=clpC PE=3 SV=1 | ATP-dependent helicase Lhr and Lhr-like helicase |
| P30015 | Probable ATP-dependent helicase lhr OS=Escherichia coli (strain K12) GN=lhr PE=3 SV=2 | ATP-dependent helicase Lhr and Lhr-like helicase |
| P55418 | Probable conjugal transfer protein TraA OS=Sinorhizobium fredii (strain NBRC 101917 / NGR234) GN=traA PE=3 SV=1 | Dtr system oriT relaxase |
| O67124 | Probable DNA double-strand break repair Rad50 ATPase OS=Aquifex aeolicus (strain VF5) GN=rad50 PE=3 SV=1 | DNA repair protein SbcC/Rad50 |
| A4IMR6 | Probable malate:quinone oxidoreductase OS=Geobacillus thermodenitrificans (strain NG80-2) GN=mqo PE=3 SV=1 | malate dehydrogenase (quinone) |
| Q7MGF9 | Probable transcriptional regulatory protein VVA0010 OS=Vibrio vulnificus (strain YJ016) GN=VVA0010 PE=3 SV=1 | uncharacterized conserved protein |
| P44970 | Probable transferrin-binding protein 1 OS=Haemophilus influenzae (strain ATCC 51907 / DSM 11121 / KW20 / Rd) GN=tbpA PE=3 SV=1 | hemoglobin/transferrin/lactoferrin receptor protein |
| Q18CD2 | Proline--tRNA ligase 1 OS=Peptoclostridium difficile (strain 630) GN=proS1 PE=3 SV=1 | prolyl-tRNA synthetase |
| A4J927 | Protein translocase subunit SecA OS=Desulfotomaculum reducens (strain MI-1) GN=secA PE=3 SV=1 | preprotein translocase subunit SecA |
| Q92JF7 | Putative surface cell antigen sca2 OS=Rickettsia conorii (strain ATCC VR-613 / Malish 7) GN=sca2 PE=1 SV=1 | 190 kD antigen precursor |
| Q8NUV0 | Putative surface protein MW2416 OS=Staphylococcus aureus (strain MW2) GN=MW2416 PE=3 SV=1 | surface protein G |
| Q2P6C7 | Pyridoxine/pyridoxamine 5'-phosphate oxidase OS=Xanthomonas oryzae pv. oryzae (strain MAFF 311018) GN=pdxH PE=3 SV=1 | pyridoxamine 5'-phosphate oxidase |
| O67037 | Reverse gyrase 1 OS=Aquifex aeolicus (strain VF5) GN=rgy1 PE=3 SV=1 | reverse gyrase |
| Q1QEP4 | Ribosome-binding factor A OS=Psychrobacter cryohalolentis (strain K5) GN=rbfA PE=3 SV=1 | ribosome-binding factor A |
| Q1GRQ3 | Ribosome-recycling factor OS=Sphingopyxis alaskensis (strain DSM 13593 / LMG 18877 / RB2256) GN=frr PE=3 SV=1 | ribosome recycling factor |
| A1JJK1 | Secretion monitor OS=Yersinia enterocolitica serotype O:8 / biotype 1B (strain NCTC 13174 / 8081) GN=secM PE=3 SV=1 | secretion monitor |
| Q9HTE9 | Serine hydroxymethyltransferase 1 OS=Pseudomonas aeruginosa (strain ATCC 15692 / DSM 22644 / CIP 104116 / JCM 14847 / LMG 12228 / 1C / PRS 101 / PAO1) GN=glyA1 PE=3 SV=1 | dol00260 Glycine, serine and threonine metabolism  dol00630 Glyoxylate and dicarboxylate metabolism  pae00460 Cyanoamino acid metabolism  dol00670 One carbon pool by folate  dol00680 Methane metabolism  dol01100 Metabolic pathways  dol01110 Biosynthesis of secondary metabolites  dol01120 Microbial metabolism in diverse environments  dol01130 Biosynthesis of antibiotics  dol01200 Carbon metabolism  dol01230 Biosynthesis of amino acids |
| Q983B6 | Serine hydroxymethyltransferase 1 OS=Rhizobium loti (strain MAFF303099) GN=glyA1 PE=3 SV=1 | dol00260 Glycine, serine and threonine metabolism  dol00630 Glyoxylate and dicarboxylate metabolism  pae00460 Cyanoamino acid metabolism  dol00670 One carbon pool by folate  dol00680 Methane metabolism  dol01100 Metabolic pathways  dol01110 Biosynthesis of secondary metabolites  dol01120 Microbial metabolism in diverse environments  dol01130 Biosynthesis of antibiotics  dol01200 Carbon metabolism  dol01230 Biosynthesis of amino acids |
| Q9KTG1 | Serine hydroxymethyltransferase 1 OS=Vibrio cholerae serotype O1 (strain ATCC 39315 / El Tor Inaba N16961) GN=glyA1 PE=3 SV=2 | dol00260 Glycine, serine and threonine metabolism  dol00630 Glyoxylate and dicarboxylate metabolism  pae00460 Cyanoamino acid metabolism  dol00670 One carbon pool by folate  dol00680 Methane metabolismMetabolic pathways  dol01110 Biosynthesis of secondary metabolites  dol01120 Microbial metabolism in diverse environments  dol01130 Biosynthesis of antibiotics  dol01200 Carbon metabolism  dol01230 Biosynthesis of amino acids |
| A1TRH1 | Serine hydroxymethyltransferase OS=Acidovorax citrulli (strain AAC00-1) GN=glyA PE=3 SV=1 | dol00260 Glycine, serine and threonine metabolism  dol00630 Glyoxylate and dicarboxylate metabolism  pae00460 Cyanoamino acid metabolism  dol00670 One carbon pool by folate  dol00680 Methane metabolism  dol01100 Metabolic pathways  dol01110 Biosynthesis of secondary metabolites  dol01120 Microbial metabolism in diverse environments  dol01130 Biosynthesis of antibiotics  dol01200 Carbon metabolism  dol01230 Biosynthesis of amino acids |
| Q6FA66 | Serine hydroxymethyltransferase OS=Acinetobacter baylyi (strain ATCC 33305 / BD413 / ADP1) GN=glyA PE=3 SV=1 | dol00260 Glycine, serine and threonine metabolism  dol00630 Glyoxylate and dicarboxylate metabolism  pae00460 Cyanoamino acid metabolism  dol00670 One carbon pool by folate  dol00680 Methane metabolism  dol01100 Metabolic pathways  dol01110 Biosynthesis of secondary metabolites  dol01120 Microbial metabolism in diverse environments  dol01130 Biosynthesis of antibiotics  dol01200 Carbon metabolism  dol01230 Biosynthesis of amino acids |
| A8MGL7 | Serine hydroxymethyltransferase OS=Alkaliphilus oremlandii (strain OhILAs) GN=glyA PE=3 SV=1 | dol00260 Glycine, serine and threonine metabolism  dol00630 Glyoxylate and dicarboxylate metabolism  pae00460 Cyanoamino acid metabolism  dol00670 One carbon pool by folate  dol00680 Methane metabolism  dol01100 Metabolic pathways  dol01110 Biosynthesis of secondary metabolites  dol01120 Microbial metabolism in diverse environments  dol01130 Biosynthesis of antibiotics  dol01200 Carbon metabolism  dol01230 Biosynthesis of amino acids |
| Q5P7P1 | Serine hydroxymethyltransferase OS=Aromatoleum aromaticum (strain EbN1) GN=glyA PE=3 SV=1 | dol00260 Glycine, serine and threonine metabolism  dol00630 Glyoxylate and dicarboxylate metabolism  pae00460 Cyanoamino acid metabolism  dol00670 One carbon pool by folate  dol00680 Methane metabolism  dol01100 Metabolic pathways  dol01110 Biosynthesis of secondary metabolites  dol01120 Microbial metabolism in diverse environments  dol01130 Biosynthesis of antibiotics  dol01200 Carbon metabolism  dol01230 Biosynthesis of amino acids |
| C1DEQ3 | Serine hydroxymethyltransferase OS=Azotobacter vinelandii (strain DJ / ATCC BAA-1303) GN=glyA PE=3 SV=1 | dol00260 Glycine, serine and threonine metabolism  dol00630 Glyoxylate and dicarboxylate metabolism  pae00460 Cyanoamino acid metabolism  dol00670 One carbon pool by folate  dol00680 Methane metabolism  dol01100 Metabolic pathways  dol01110 Biosynthesis of secondary metabolites  dol01120 Microbial metabolism in diverse environments  dol01130 Biosynthesis of antibiotics  dol01200 Carbon metabolism  dol01230 Biosynthesis of amino acids |
| A6L5K3 | Serine hydroxymethyltransferase OS=Bacteroides vulgatus (strain ATCC 8482 / DSM 1447 / JCM 5826 / NBRC 14291 / NCTC 11154) GN=glyA PE=3 SV=1 | dol00260 Glycine, serine and threonine metabolism  dol00630 Glyoxylate and dicarboxylate metabolism  pae00460 Cyanoamino acid metabolism  dol00670 One carbon pool by folate  dol00680 Methane metabolism  dol01100 Metabolic pathways  dol01110 Biosynthesis of secondary metabolites  dol01120 Microbial metabolism in diverse environments  dol01130 Biosynthesis of antibiotics  dol01200 Carbon metabolism  dol01230 Biosynthesis of amino acids |
| Q6MLK1 | Serine hydroxymethyltransferase OS=Bdellovibrio bacteriovorus (strain ATCC 15356 / DSM 50701 / NCIB 9529 / HD100) GN=glyA PE=3 SV=1 | dol00260 Glycine, serine and threonine metabolism  dol00630 Glyoxylate and dicarboxylate metabolism  pae00460 Cyanoamino acid metabolism  dol00670 One carbon pool by folate  dol00680 Methane metabolism  dol01100 Metabolic pathways  dol01110 Biosynthesis of secondary metabolites  dol01120 Microbial metabolism in diverse environments  dol01130 Biosynthesis of antibiotics  dol01200 Carbon metabolism  dol01230 Biosynthesis of amino acids |
| Q492D5 | Serine hydroxymethyltransferase OS=Blochmannia pennsylvanicus (strain BPEN) GN=glyA PE=3 SV=1 | dol00260 Glycine, serine and threonine metabolism  dol00630 Glyoxylate and dicarboxylate metabolism  pae00460 Cyanoamino acid metabolism  dol00670 One carbon pool by folate  dol00680 Methane metabolism  dol01100 Metabolic pathways  dol01110 Biosynthesis of secondary metabolites  dol01120 Microbial metabolism in diverse environments  dol01130 Biosynthesis of antibiotics  dol01200 Carbon metabolism  dol01230 Biosynthesis of amino acids |
| Q2KV15 | Serine hydroxymethyltransferase OS=Bordetella avium (strain 197N) GN=glyA PE=3 SV=1 | dol00260 Glycine, serine and threonine metabolism  dol00630 Glyoxylate and dicarboxylate metabolism  pae00460 Cyanoamino acid metabolism  dol00670 One carbon pool by folate  dol00680 Methane metabolism  dol01100 Metabolic pathways  dol01110 Biosynthesis of secondary metabolites  dol01120 Microbial metabolism in diverse environments  dol01130 Biosynthesis of antibiotics  dol01200 Carbon metabolism  dol01230 Biosynthesis of amino acids |
| B3EFN5 | Serine hydroxymethyltransferase OS=Chlorobium limicola (strain DSM 245 / NBRC 103803 / 6330) GN=glyA PE=3 SV=2 | dol00260 Glycine, serine and threonine metabolism  dol00630 Glyoxylate and dicarboxylate metabolism  pae00460 Cyanoamino acid metabolism  dol00670 One carbon pool by folate  dol00680 Methane metabolism  dol01100 Metabolic pathways  dol01110 Biosynthesis of secondary metabolites  dol01120 Microbial metabolism in diverse environments  dol01130 Biosynthesis of antibiotics  dol01200 Carbon metabolism  dol01230 Biosynthesis of amino acids |
| B3EMW0 | Serine hydroxymethyltransferase OS=Chlorobium phaeobacteroides (strain BS1) GN=glyA PE=3 SV=2 | dol00260 Glycine, serine and threonine metabolism  dol00630 Glyoxylate and dicarboxylate metabolism  pae00460 Cyanoamino acid metabolism  dol00670 One carbon pool by folate  dol00680 Methane metabolism  dol01100 Metabolic pathways  dol01110 Biosynthesis of secondary metabolites  dol01120 Microbial metabolism in diverse environments  dol01130 Biosynthesis of antibiotics  dol01200 Carbon metabolism  dol01230 Biosynthesis of amino acids |
| A4SFY3 | Serine hydroxymethyltransferase OS=Chlorobium phaeovibrioides (strain DSM 265 / 1930) GN=glyA PE=3 SV=1 | dol00260 Glycine, serine and threonine metabolism  dol00630 Glyoxylate and dicarboxylate metabolism  pae00460 Cyanoamino acid metabolism  dol00670 One carbon pool by folate  dol00680 Methane metabolism  dol01100 Metabolic pathways  dol01110 Biosynthesis of secondary metabolites  dol01120 Microbial metabolism in diverse environments  dol01130 Biosynthesis of antibiotics  dol01200 Carbon metabolism  dol01230 Biosynthesis of amino acids |
| B3QUG2 | Serine hydroxymethyltransferase OS=Chloroherpeton thalassium (strain ATCC 35110 / GB-78) GN=glyA PE=3 SV=1 | dol00260 Glycine, serine and threonine metabolism  dol00630 Glyoxylate and dicarboxylate metabolism  pae00460 Cyanoamino acid metabolism  dol00670 One carbon pool by folate  dol00680 Methane metabolism  dol01100 Metabolic pathways  dol01110 Biosynthesis of secondary metabolites  dol01120 Microbial metabolism in diverse environments  dol01130 Biosynthesis of antibiotics  dol01200 Carbodn metabolism  dol01230 Biosynthesis of amino acids |
| Q47IH1 | Serine hydroxymethyltransferase OS=Dechloromonas aromatica (strain RCB) GN=glyA PE=3 SV=1 | dol00260 Glycine, serine and threonine metabolism  dol00630 Glyoxylate and dicarboxylate metabolism  pae00460 Cyanoamino acid metabolism  dol00670 One carbon pool by folate  dol00680 Methane metabolism  dol01100 Metabolic pathways  dol01110 Biosynthesis of secondary metabolites  dol01120 Microbial metabolism in diverse environments  dol01130 Biosynthesis of antibiotics  dol01200 Carbon metabolism  dol01230 Biosynthesis of amino acids |
| Q1J1W0 | Serine hydroxymethyltransferase OS=Deinococcus geothermalis (strain DSM 11300) GN=glyA PE=3 SV=1 | dol00260 Glycine, serine and threonine metabolism  dol00630 Glyoxylate and dicarboxylate metabolism  pae00460 Cyanoamino acid metabolism  dol00670 One carbon pool by folate  dol00680 Methane metabolism  dol01100 Metabolic pathways  dol01110 Biosynthesis of secondary metabolites  dol01120 Microbial metabolism in diverse environments  dol01130 Biosynthesis of antibiotics  dol01200 Carbon metabolism  dol01230 Biosynthesis of amino acids |
| Q9RYB2 | Serine hydroxymethyltransferase OS=Deinococcus radiodurans (strain ATCC 13939 / DSM 20539 / JCM 16871 / LMG 4051 / NBRC 15346 / NCIMB 9279 / R1 / VKM B-1422) GN=glyA PE=3 SV=2 | dol00260 Glycine, serine and threonine metabolism  dol00630 Glyoxylate and dicarboxylate metabolism  pae00460 Cyanoamino acid metabolism  dol00670 One carbon pool by folate  dol00680 Methane metabolism  dol01100 Metabolic pathways  dol01110 Biosynthesis of secondary metabolites  dol01120 Microbial metabolism in diverse environments  dol01130 Biosynthesis of antibiotics  dol01200 Carbon metabolism  dol01230 Biosynthesis of amino acids |
| A8ZTV3 | Serine hydroxymethyltransferase OS=Desulfococcus oleovorans (strain DSM 6200 / Hxd3) GN=glyA PE=3 SV=1 | dol00260 Glycine, serine and threonine metabolism  dol00630 Glyoxylate and dicarboxylate metabolism  pae00460 Cyanoamino acid metabolism  dol00670 One carbon pool by folate  dol00680 Methane metabolism  dol01100 Metabolic pathways  dol01110 Biosynthesis of secondary metabolites  dol01120 Microbial metabolism in diverse environments  dol01130 Biosynthesis of antibiotics  dol01200 Carbon metabolism  dol01230 Biosynthesis of amino acids |
| Q3YRD1 | Serine hydroxymethyltransferase OS=Ehrlichia canis (strain Jake) GN=glyA PE=3 SV=1 | dol00260 Glycine, serine and threonine metabolism  dol00630 Glyoxylate and dicarboxylate metabolism  pae00460 Cyanoamino acid metabolism  dol00670 One carbon pool by folate  dol00680 Methane metabolism  dol01100 Metabolic pathways  dol01110 Biosynthesis of secondary metabolites  dol01120 Microbial metabolism in diverse environments  dol01130 Biosynthesis of antibiotics  dol01200 Carbon metabolism  dol01230 Biosynthesis of amino acids |
| Q2NAR9 | Serine hydroxymethyltransferase OS=Erythrobacter litoralis (strain HTCC2594) GN=glyA PE=3 SV=1 | dol00260 Glycine, serine and threonine metabolism  dol00630 Glyoxylate and dicarboxylate metabolism  pae00460 Cyanoamino acid metabolism  dol00670 One carbon pool by folate  dol00680 Methane metabolism  dol01100 Metabolic pathways  dol01110 Biosynthesis of secondary metabolites  dol01120 Microbial metabolism in diverse environments  dol01130 Biosynthesis of antibiotics  dol01200 Carbon metabolism  dol01230 Biosynthesis of amino acids |
| A6GXG2 | Serine hydroxymethyltransferase OS=Flavobacterium psychrophilum (strain JIP02/86 / ATCC 49511) GN=glyA PE=3 SV=1 | dol00260 Glycine, serine and threonine metabolism  dol00630 Glyoxylate and dicarboxylate metabolism  pae00460 Cyanoamino acid metabolism  dol00670 One carbon pool by folate  dol00680 Methane metabolism  dol01100 Metabolic pathways  dol01110 Biosynthesis of secondary metabolites  dol01120 Microbial metabolism in diverse environments  dol01130 Biosynthesis of antibiotics  dol01200 Carbon metabolism  dol01230 Biosynthesis of amino acids |
| B9M0W5 | Serine hydroxymethyltransferase OS=Geobacter daltonii (strain DSM 22248 / JCM 15807 / FRC-32) GN=glyA PE=3 SV=1 | dol00260 Glycine, serine and threonine metabolism  dol00630 Glyoxylate and dicarboxylate metabolism  pae00460 Cyanoamino acid metabolism  dol00670 One carbon pool by folate  dol00680 Methane metabolism  dol01100 Metabolic pathways  dol01110 Biosynthesis of secondary metabolites  dol01120 Microbial metabolism in diverse environments  dol01130 Biosynthesis of antibiotics  dol01200 Carbon metabolism  dol01230 Biosynthesis of amino acids |
| C6E348 | Serine hydroxymethyltransferase OS=Geobacter sp. (strain M21) GN=glyA PE=3 SV=1 | dol00260 Glycine, serine and threonine metabolism  dol00630 Glyoxylate and dicarboxylate metabolism  pae00460 Cyanoamino acid metabolism  dol00670 One carbon pool by folate  dol00680 Methane metabolism  dol01100 Metabolic pathways  dol01110 Biosynthesis of secondary metabolites  dol01120 Microbial metabolism in diverse environments  dol01130 Biosynthesis of antibiotics  dol01200 Carbon metabolism  dol01230 Biosynthesis of amino acids |
| A5GF66 | Serine hydroxymethyltransferase OS=Geobacter uraniireducens (strain Rf4) GN=glyA PE=3 SV=1 | dol00260 Glycine, serine and threonine metabolism  dol00630 Glyoxylate and dicarboxylate metabolism  pae00460 Cyanoamino acid metabolism  dol00670 One carbon pool by folate  dol00680 Methane metabolism  dol01100 Metabolic pathways  dol01110 Biosynthesis of secondary metabolites  dol01120 Microbial metabolism in diverse environments  dol01130 Biosynthesis of antibiotics  dol01200 Carbon metabolism  dol01230 Biosynthesis of amino acids |
| A0M3N2 | Serine hydroxymethyltransferase OS=Gramella forsetii (strain KT0803) GN=glyA PE=3 SV=1 | dol00260 Glycine, serine and threonine metabolism  dol00630 Glyoxylate and dicarboxylate metabolism  pae00460 Cyanoamino acid metabolism  dol00670 One carbon pool by folate  dol00680 Methane metabolism  dol01100 Metabolic pathways  dol01110 Biosynthesis of secondary metabolites  dol01120 Microbial metabolism in diverse environments  dol01130 Biosynthesis of antibiotics  dol01200 Carbon metabolism  dol01230 Biosynthesis of amino acids |
| A2RIS0 | Serine hydroxymethyltransferase OS=Lactococcus lactis subsp. cremoris (strain MG1363) GN=glyA PE=3 SV=1 | dol00260 Glycine, serine and threonine metabolism  dol00630 Glyoxylate and dicarboxylate metabolism  pae00460 Cyanoamino acid metabolism  dol00670 One carbon pool by folate  dol00680 Methane metabolism  dol01100 Metabolic pathways  dol01110 Biosynthesis of secondary metabolites  dol01120 Microbial metabolism in diverse environments  dol01130 Biosynthesis of antibiotics  dol01200 Carbon metabolism  dol01230 Biosynthesis of amino acids |
| B1HM45 | Serine hydroxymethyltransferase OS=Lysinibacillus sphaericus (strain C3-41) GN=glyA PE=3 SV=1 | dol00260 Glycine, serine and threonine metabolism  dol00630 Glyoxylate and dicarboxylate metabolism  pae00460 Cyanoamino acid metabolism  dol00670 One carbon pool by folate  dol00680 Methane metabolism  dol01100 Metabolic pathways  dol01110 Biosynthesis of secondary metabolites  dol01120 Microbial metabolism in diverse environments  dol01130 Biosynthesis of antibiotics  dol01200 Carbon metabolism  dol01230 Biosynthesis of amino acids |
| Q1H003 | Serine hydroxymethyltransferase OS=Methylobacillus flagellatus (strain KT / ATCC 51484 / DSM 6875) GN=glyA PE=3 SV=2 | dol00260 Glycine, serine and threonine metabolism  dol00630 Glyoxylate and dicarboxylate metabolism  pae00460 Cyanoamino acid metabolism  dol00670 One carbon pool by folate  dol00680 Methane metabolism  dol01100 Metabolic pathways  dol01110 Biosynthesis of secondary metabolites  dol01120 Microbial metabolism in diverse environments  dol01130 Biosynthesis of antibiotics  dol01200 Carbon metabolism  dol01230 Biosynthesis of amino acids |
| Q6MS85 | Serine hydroxymethyltransferase OS=Mycoplasma mycoides subsp. mycoides SC (strain PG1) GN=glyA PE=3 SV=1 | dol00260 Glycine, serine and threonine metabolism  dol00630 Glyoxylate and dicarboxylate metabolism  pae00460 Cyanoamino acid metabolism  dol00670 One carbon pool by folate  dol00680 Methane metabolism  dol01100 Metabolic pathways  dol01110 Biosynthesis of secondary metabolites  dol01120 Microbial metabolism in diverse environments  dol01130 Biosynthesis of antibiotics  dol01200 Carbon metabolism  dol01230 Biosynthesis of amino acids |
| Q2GEI3 | Serine hydroxymethyltransferase OS=Neorickettsia sennetsu (strain ATCC VR-367 / Miyayama) GN=glyA PE=3 SV=1 | dol00260 Glycine, serine and threonine metabolism  dol00630 Glyoxylate and dicarboxylate metabolism  pae00460 Cyanoamino acid metabolism  dol00670 One carbon pool by folate  dol00680 Methane metabolism  dol01100 Metabolic pathways  dol01110 Biosynthesis of secondary metabolites  dol01120 Microbial metabolism in diverse environments  dol01130 Biosynthesis of antibiotics  dol01200 Carbon metabolism  dol01230 Biosynthesis of amino acids |
| A6LBG7 | Serine hydroxymethyltransferase OS=Parabacteroides distasonis (strain ATCC 8503 / DSM 20701 / CIP 104284 / JCM 5825 / NCTC 11152) GN=glyA PE=3 SV=1 | dol00260 Glycine, serine and threonine metabolism  dol00630 Glyoxylate and dicarboxylate metabolism  pae00460 Cyanoamino acid metabolism  dol00670 One carbon pool by folate  dol00680 Methane metabolism  dol01100 Metabolic pathways  dol01110 Biosynthesis of secondary metabolites  dol01120 Microbial metabolism in diverse environments  dol01130 Biosynthesis of antibiotics  dol01200 Carbon metabolism  dol01230 Biosynthesis of amino acids |
| P57830 | Serine hydroxymethyltransferase OS=Pasteurella multocida (strain Pm70) GN=glyA PE=3 SV=1 | dol00260 Glycine, serine and threonine metabolism  dol00630 Glyoxylate and dicarboxylate metabolism  pae00460 Cyanoamino acid metabolism  dol00670 One carbon pool by folate  dol00680 Methane metabolism  dol01100 Metabolic pathways  dol01110 Biosynthesis of secondary metabolites  dol01120 Microbial metabolism in diverse environments  dol01130 Biosynthesis of antibiotics  dol01200 Carbon metabolism  dol01230 Biosynthesis of amino acids |
| Q03EK4 | Serine hydroxymethyltransferase OS=Pediococcus pentosaceus (strain ATCC 25745 / CCUG 21536 / LMG 10740 / 183-1w) GN=glyA PE=3 SV=1 | dol00260 Glycine, serine and threonine metabolism  dol00630 Glyoxylate and dicarboxylate metabolism  pae00460 Cyanoamino acid metabolism  dol00670 One carbon pool by folate  dol00680 Methane metabolism  dol01100 Metabolic pathways  dol01110 Biosynthesis of secondary metabolites  dol01120 Microbial metabolism in diverse environments  dol01130 Biosynthesis of antibiotics  dol01200 Carbon metabolism  dol01230 Biosynthesis of amino acids |
| Q4FLT4 | Serine hydroxymethyltransferase OS=Pelagibacter ubique (strain HTCC1062) GN=glyA PE=3 SV=1 | dol00260 Glycine, serine and threonine metabolism  dol00630 Glyoxylate and dicarboxylate metabolism  pae00460 Cyanoamino acid metabolism  dol00670 One carbon pool by folate  dol00680 Methane metabolism  dol01100 Metabolic pathways  dol01110 Biosynthesis of secondary metabolites  dol01120 Microbial metabolism in diverse environments  dol01130 Biosynthesis of antibiotics  dol01200 Carbon metabolism  dol01230 Biosynthesis of amino acids |
| B4SE31 | Serine hydroxymethyltransferase OS=Pelodictyon phaeoclathratiforme (strain DSM 5477 / BU-1) GN=glyA PE=3 SV=1 | dol00260 Glycine, serine and threonine metabolism  dol00630 Glyoxylate and dicarboxylate metabolism  pae00460 Cyanoamino acid metabolism  dol00670 One carbon pool by folate  dol00680 Methane metabolism  dol01100 Metabolic pathways  dol01110 Biosynthesis of secondary metabolites  dol01120 Microbial metabolism in diverse environments  dol01130 Biosynthesis of antibiotics  dol01200 Carbon metabolism  dol01230 Biosynthesis of amino acids |
| B4RB35 | Serine hydroxymethyltransferase OS=Phenylobacterium zucineum (strain HLK1) GN=glyA PE=3 SV=1 | dol00260 Glycine, serine and threonine metabolism  dol00630 Glyoxylate and dicarboxylate metabolism  pae00460 Cyanoamino acid metabolism  dol00670 One carbon pool by folate  dol00680 Methane metabolism  dol01100 Metabolic pathways  dol01110 Biosynthesis of secondary metabolites  dol01120 Microbial metabolism in diverse environments  dol01130 Biosynthesis of antibiotics  dol01200 Carbon metabolism  dol01230 Biosynthesis of amino acids |
| Q4FUZ8 | Serine hydroxymethyltransferase OS=Psychrobacter arcticus (strain DSM 17307 / 273-4) GN=glyA PE=3 SV=1 | dol00260 Glycine, serine and threonine metabolism  dol00630 Glyoxylate and dicarboxylate metabolism  pae00460 Cyanoamino acid metabolism  dol00670 One carbon pool by folate  dol00680 Methane metabolism  dol01100 Metabolic pathways  dol01110 Biosynthesis of secondary metabolites  dol01120 Microbial metabolism in diverse environments  dol01130 Biosynthesis of antibiotics  dol01200 Carbon metabolism  dol01230 Biosynthesis of amino acids |
| Q1MIU5 | Serine hydroxymethyltransferase OS=Rhizobium leguminosarum bv. viciae (strain 3841) GN=glyA PE=3 SV=1 | dol00260 Glycine, serine and threonine metabolism  dol00630 Glyoxylate and dicarboxylate metabolism  pae00460 Cyanoamino acid metabolism  dol00670 One carbon pool by folate  dol00680 Methane metabolism  dol01100 Metabolic pathways  dol01110 Biosynthesis of secondary metabolites  dol01120 Microbial metabolism in diverse environments  dol01130 Biosynthesis of antibiotics  dol01200 Carbon metabolism  dol01230 Biosynthesis of amino acids |
| Q21NP8 | Serine hydroxymethyltransferase OS=Saccharophagus degradans (strain 2-40 / ATCC 43961 / DSM 17024) GN=glyA PE=3 SV=1 | dol00260 Glycine, serine and threonine metabolism  dol00630 Glyoxylate and dicarboxylate metabolism  pae00460 Cyanoamino acid metabolism  dol00670 One carbon pool by folate  dol00680 Methane metabolism  dol01100 Metabolic pathways  dol01110 Biosynthesis of secondary metabolites  dol01120 Microbial metabolism in diverse environments  dol01130 Biosynthesis of antibiotics  dol01200 Carbon metabolism  dol01230 Biosynthesis of amino acids |
| B8E6W1 | Serine hydroxymethyltransferase OS=Shewanella baltica (strain OS223) GN=glyA PE=3 SV=1 | dol00260 Glycine, serine and threonine metabolism  dol00630 Glyoxylate and dicarboxylate metabolism  pae00460 Cyanoamino acid metabolism  dol00670 One carbon pool by folate  dol00680 Methane metabolism  dol01100 Metabolic pathways  dol01110 Biosynthesis of secondary metabolites  dol01120 Microbial metabolism in diverse environments  dol01130 Biosynthesis of antibiotics  dol01200 Carbon metabolism  dol01230 Biosynthesis of amino acids |
| B0TJY5 | Serine hydroxymethyltransferase OS=Shewanella halifaxensis (strain HAW-EB4) GN=glyA PE=3 SV=1 | dol00260 Glycine, serine and threonine metabolism  dol00630 Glyoxylate and dicarboxylate metabolism  pae00460 Cyanoamino acid metabolism  dol00670 One carbon pool by folate  dol00680 Methane metabolism  dol01100 Metabolic pathways  dol01110 Biosynthesis of secondary metabolites  dol01120 Microbial metabolism in diverse environments  dol01130 Biosynthesis of antibiotics  dol01200 Carbon metabolism  dol01230 Biosynthesis of amino acids |
| A5V5D1 | Serine hydroxymethyltransferase OS=Sphingomonas wittichii (strain RW1 / DSM 6014 / JCM 10273) GN=glyA PE=3 SV=1 | dol00260 Glycine, serine and threonine metabolism  dol00630 Glyoxylate and dicarboxylate metabolism  pae00460 Cyanoamino acid metabolism  dol00670 One carbon pool by folate  dol00680 Methane metabolism  dol01100 Metabolic pathways  dol01110 Biosynthesis of secondary metabolites  dol01120 Microbial metabolism in diverse environments  dol01130 Biosynthesis of antibiotics  dol01200 Carbon metabolism  dol01230 Biosynthesis of amino acids |
| B9DS48 | Serine hydroxymethyltransferase OS=Streptococcus uberis (strain ATCC BAA-854 / 0140J) GN=glyA PE=3 SV=1 | dol00260 Glycine, serine and threonine metabolism  dol00630 Glyoxylate and dicarboxylate metabolism  pae00460 Cyanoamino acid metabolism  dol00670 One carbon pool by folate  dol00680 Methane metabolism  dol01100 Metabolic pathways  dol01110 Biosynthesis of secondary metabolites  dol01120 Microbial metabolism in diverse environments  dol01130 Biosynthesis of antibiotics  dol01200 Carbon metabolism  dol01230 Biosynthesis of amino acids |
| A0LI16 | Serine hydroxymethyltransferase OS=Syntrophobacter fumaroxidans (strain DSM 10017 / MPOB) GN=glyA PE=3 SV=1 | dol00260 Glycine, serine and threonine metabolism  dol00630 Glyoxylate and dicarboxylate metabolism  pae00460 Cyanoamino acid metabolism  dol00670 One carbon pool by folate  dol00680 Methane metabolism  dol01100 Metabolic pathways  dol01110 Biosynthesis of secondary metabolites  dol01120 Microbial metabolism in diverse environments  dol01130 Biosynthesis of antibiotics  dol01200 Carbon metabolism  dol01230 Biosynthesis of amino acids |
| Q2LQM6 | Serine hydroxymethyltransferase OS=Syntrophus aciditrophicus (strain SB) GN=glyA PE=3 SV=1 | dol00260 Glycine, serine and threonine metabolism  dol00630 Glyoxylate and dicarboxylate metabolism  pae00460 Cyanoamino acid metabolism  dol00670 One carbon pool by folate  dol00680 Methane metabolism  dol01100 Metabolic pathways  dol01110 Biosynthesis of secondary metabolites  dol01120 Microbial metabolism in diverse environments  dol01130 Biosynthesis of antibiotics  dol01200 Carbon metabolism  dol01230 Biosynthesis of amino acids |
| Q8CMP4 | Serine-aspartate repeat-containing protein F OS=Staphylococcus epidermidis (strain ATCC 12228) GN=sdrF PE=3 SV=1 | Ser-Asp rich fibrinogen-binding,bone sialoprotein-binding protein |
| Q7N8R3 | Serralysin OS=Photorhabdus luminescens subsp. laumondii (strain DSM 15139 / CIP 105565 / TT01) GN=prtA PE=3 SV=1 | Cationic antimicrobial peptide (CAMP) resistance |
| O32001 | SPBc2 prophage-derived endonuclease YokF OS=Bacillus subtilis (strain 168) GN=yokF PE=1 SV=1 | endonuclease |
| P0C0I3 | Streptolysin O OS=Streptococcus pyogenes serotype M1 GN=slo PE=3 SV=1 | spy02024 Quorum sensing |
| Q2G2B2 | Surface protein G OS=Staphylococcus aureus (strain NCTC 8325) GN=sasG PE=1 SV=1 | sao05150 Staphylococcus aureus infection |
| P38537 | Surface-layer 125 kDa protein OS=Lysinibacillus sphaericus PE=3 SV=1 | Coating of bacterial surface |
| C1DF42 | Threonine--tRNA ligase OS=Azotobacter vinelandii (strain DJ / ATCC BAA-1303) GN=thrS PE=3 SV=1 | avn00970 Aminoacyl-tRNA biosynthesis |
| B3DRX5 | Threonine--tRNA ligase OS=Bifidobacterium longum (strain DJO10A) GN=thrS PE=3 SV=1 | blj00970 Aminoacyl-tRNA biosynthesis |
| Q2L2M5 | Threonine--tRNA ligase OS=Bordetella avium (strain 197N) GN=thrS PE=3 SV=1 | bav00970 Aminoacyl-tRNA biosynthesis |
| A1VXT5 | Threonine--tRNA ligase OS=Campylobacter jejuni subsp. jejuni serotype O:23/36 (strain 81-176) GN=thrS PE=3 SV=1 | cjj00970 Aminoacyl-tRNA biosynthesis |
| B9KEB1 | Threonine--tRNA ligase OS=Campylobacter lari (strain RM2100 / D67 / ATCC BAA-1060) GN=thrS PE=3 SV=1 | cla00970 Aminoacyl-tRNA biosynthesis |
| B8GZS7 | Threonine--tRNA ligase OS=Caulobacter crescentus (strain NA1000 / CB15N) GN=thrS PE=3 SV=1 | ccs00970 Aminoacyl-tRNA biosynthesis |
| B0T0Z7 | Threonine--tRNA ligase OS=Caulobacter sp. (strain K31) GN=thrS PE=3 SV=1 | cak00970 Aminoacyl-tRNA biosynthesis |
| Q0KBZ3 | Threonine--tRNA ligase OS=Cupriavidus necator (strain ATCC 17699 / H16 / DSM 428 / Stanier 337) GN=thrS PE=3 SV=1 | reh00970 Aminoacyl-tRNA biosynthesis |
| C5B846 | Threonine--tRNA ligase OS=Edwardsiella ictaluri (strain 93-146) GN=thrS PE=3 SV=1 | eic00970 Aminoacyl-tRNA biosynthesis |
| Q2N6X5 | Threonine--tRNA ligase OS=Erythrobacter litoralis (strain HTCC2594) GN=thrS PE=3 SV=1 | eli00970 Aminoacyl-tRNA biosynthesis |
| Q5FSP9 | Threonine--tRNA ligase OS=Gluconobacter oxydans (strain 621H) GN=thrS PE=3 SV=1 | gox00970 Aminoacyl-tRNA biosynthesis |
| A4G619 | Threonine--tRNA ligase OS=Herminiimonas arsenicoxydans GN=thrS PE=3 SV=1 | har00970 Aminoacyl-tRNA biosynthesis |
| B5XQC6 | Threonine--tRNA ligase OS=Klebsiella pneumoniae (strain 342) GN=thrS PE=3 SV=1 | kpe00970 Aminoacyl-tRNA biosynthesis |
| Q2WAI1 | Threonine--tRNA ligase OS=Magnetospirillum magneticum (strain AMB-1 / ATCC 700264) GN=thrS PE=3 SV=1 | mag00970 Aminoacyl-tRNA biosynthesis |
| A5CD12 | Threonine--tRNA ligase OS=Orientia tsutsugamushi (strain Boryong) GN=thrS PE=3 SV=1 | ots00970 Aminoacyl-tRNA biosynthesis |
| Q6D4G8 | Threonine--tRNA ligase OS=Pectobacterium atrosepticum (strain SCRI 1043 / ATCC BAA-672) GN=thrS PE=3 SV=1 | eca00970 Aminoacyl-tRNA biosynthesis |
| A2BQ78 | Threonine--tRNA ligase OS=Prochlorococcus marinus (strain AS9601) GN=thrS PE=3 SV=1 | pmb00970 Aminoacyl-tRNA biosynthesis |
| A2CAU4 | Threonine--tRNA ligase OS=Prochlorococcus marinus (strain MIT 9303) GN=thrS PE=3 SV=1 | pmf00970 Aminoacyl-tRNA biosynthesis |
| Q4FPW3 | Threonine--tRNA ligase OS=Psychrobacter arcticus (strain DSM 17307 / 273-4) GN=thrS PE=3 SV=1 | par00970 Aminoacyl-tRNA biosynthesis |
| Q8XZ29 | Threonine--tRNA ligase OS=Ralstonia solanacearum (strain GMI1000) GN=thrS PE=3 SV=1 | rso00970 Aminoacyl-tRNA biosynthesis |
| A3PLC6 | Threonine--tRNA ligase OS=Rhodobacter sphaeroides (strain ATCC 17029 / ATH 2.4.9) GN=thrS PE=3 SV=1 | sde00970  Aminoacyl-tRNA biosynthesis |
| Q21KE0 | Threonine--tRNA ligase OS=Saccharophagus degradans (strain 2-40 / ATCC 43961 / DSM 17024) GN=thrS PE=3 SV=1 | sgl00970  Aminoacyl-tRNA biosynthesis |
| Q2NT31 | Threonine--tRNA ligase OS=Sodalis glossinidius (strain morsitans) GN=thrS PE=3 SV=1 | sun00970 Aminoacyl-tRNA biosynthesis |
| A6QD00 | Threonine--tRNA ligase OS=Sulfurovum sp. (strain NBC37-1) GN=thrS PE=3 SV=1 | genetic information processing |
| Q01996 | Transferrin-binding protein 1 OS=Neisseria gonorrhoeae GN=tbp1 PE=3 SV=1 | genetic information processing |
| B8J1Y4 | Translation initiation factor IF-2 OS=Desulfovibrio desulfuricans (strain ATCC 27774 / DSM 6949) GN=infB PE=3 SV=1 | genetic information processing |
| Q72ER1 | Translation initiation factor IF-2 OS=Desulfovibrio vulgaris (strain Hildenborough / ATCC 29579 / NCIMB 8303) GN=infB PE=3 SV=1 | genetic information processing |
| Q74CT3 | Translation initiation factor IF-2 OS=Geobacter sulfurreducens (strain ATCC 51573 / DSM 12127 / PCA) GN=infB PE=3 SV=1 | genetic information processing |
| A5GF86 | Translation initiation factor IF-2 OS=Geobacter uraniireducens (strain Rf4) GN=infB PE=3 SV=1 | genetic information processing |
| B2IME4 | Translation initiation factor IF-2 OS=Streptococcus pneumoniae (strain CGSP14) GN=infB PE=3 SV=1 | genetic information processing |
| A6QBQ5 | Translation initiation factor IF-2 OS=Sulfurovum sp. (strain NBC37-1) GN=infB PE=3 SV=1 | Protein synthesis. Protects formylmethionyl-tRNA from spontaneous hydrolysis and promotes its binding to the 30S ribosomal subunits. Hydrolysis of GTP |
| P10021 | Transposase for transposon Tn4430 OS=Bacillus thuringiensis GN=tnpA PE=3 SV=1 | genetic information processing |
| B0TMW0 | tRNA 2-selenouridine synthase OS=Shewanella halifaxensis (strain HAW-EB4) GN=selU PE=3 SV=1 | genetic information processing |
| B1KKR3 | tRNA 5-methylaminomethyl-2-thiouridine biosynthesis bifunctional protein MnmC OS=Shewanella woodyi (strain ATCC 51908 / MS32) GN=mnmC PE=3 SV=1 | genetic information processing |
| Q3AU39 | tRNA-2-methylthio-N(6)-dimethylallyladenosine synthase OS=Chlorobium chlorochromatii (strain CaD3) GN=miaB PE=3 SV=1 | signaling and cellular processes |
| P08956 | Type I restriction enzyme EcoKI R protein OS=Escherichia coli (strain K12) GN=hsdR PE=3 SV=3 | DNA restriction-modification system |
| P12364 | Type III restriction-modification system EcoP15I enzyme mod OS=Escherichia coli GN=mod PE=3 SV=1 | lac00300 Lysine biosynthesis  lac00550 Peptidoglycan biosynthesis  lac01100 Metabolic pathways |
| Q5FI54 | UDP-N-acetylmuramyl-tripeptide synthetase OS=Lactobacillus acidophilus (strain ATCC 700396 / NCK56 / N2 / NCFM) GN=murE PE=3 SV=1 | lac00300 Lysine biosynthesis  lac00550 Peptidoglycan biosynthesis  lac01100 Metabolic pathways |
| A5URF5 | UDP-N-acetylmuramyl-tripeptide synthetase OS=Roseiflexus sp. (strain RS-1) GN=murE PE=3 SV=1 | cell cycle, cell wall organization, peptidoglycan biosynthetic process, regulation of cell shape |
| P55666 | Uncharacterized hydrolase/peptidase y4tL OS=Sinorhizobium fredii (strain NBRC 101917 / NGR234) GN=NGR_a01470 PE=3 SV=1 | Function unknown |
| P0A4Y3 | Uncharacterized protein Mb1353c OS=Mycobacterium bovis (strain ATCC BAA-935 / AF2122/97) GN=Mb1353c PE=3 SV=1 | cyclic nucleotide biosynthetic process, intracellular signal transduction |
| O83892 | Uncharacterized protein TP_0922 OS=Treponema pallidum (strain Nichols) GN=TP_0922 PE=4 SV=1 | Function unknown |
| Q8YRI1 | Uncharacterized WD repeat-containing protein alr3466 OS=Nostoc sp. (strain PCC 7120 / SAG 25.82 / UTEX 2576) GN=alr3466 PE=3 SV=1 | Function unknown |
| Q92HD6 | UPF0192 protein RC0835 OS=Rickettsia conorii (strain ATCC VR-613 / Malish 7) GN=RC0835 PE=3 SV=1 | Protects the bacterial cell from host peptidases |
| A6L239 | UPF0597 protein BVU_2091 OS=Bacteroides vulgatus (strain ATCC 8482 / DSM 1447 / JCM 5826 / NBRC 14291 / NCTC 11154) GN=BVU_2091 PE=3 SV=1 | Membrane protein |
| B9L7K6 | UPF0597 protein NAMH_0191 OS=Nautilia profundicola (strain ATCC BAA-1463 / DSM 18972 / AmH) GN=NAMH_0191 PE=3 SV=1 | Function unknown |
| B4U7X3 | UPF0753 protein HY04AAS1_0547 OS=Hydrogenobaculum sp. (strain Y04AAS1) GN=HY04AAS1_0547 PE=3 SV=1 | Function unknown |
| C1DU51 | UPF0753 protein SULAZ_0653 OS=Sulfurihydrogenibium azorense (strain Az-Fu1 / DSM 15241 / OCM 825) GN=SULAZ_0653 PE=3 SV=1 | bbu03420 Nucleotide excision repair |
| O51777 | UvrABC system protein A OS=Borrelia burgdorferi (strain ATCC 35210 / B31 / CIP 102532 / DSM 4680) GN=uvrA PE=3 SV=1 | SOS response, nucleotide-excision repair |
| Q50968 | UvrABC system protein A OS=Neisseria gonorrhoeae GN=uvrA PE=3 SV=1 | nma03420 Nucleotide excision repair |
| Q9JUS4 | UvrABC system protein A OS=Neisseria meningitidis serogroup A / serotype 4A (strain Z2491) GN=uvrA PE=3 SV=1 | nme03420Nucleotide excision repair |
| Q9JZP1 | UvrABC system protein A OS=Neisseria meningitidis serogroup B (strain MC58) GN=uvrA PE=3 SV=1 | ate03420 Nucleotide excision repair |
| B9MS82 | UvrABC system protein B OS=Caldicellulosiruptor bescii (strain ATCC BAA-1888 / DSM 6725 / Z-1320) GN=uvrB PE=3 SV=1 | deh03420 Nucleotide excision repair |
| Q3ZZK7 | UvrABC system protein B OS=Dehalococcoides mccartyi (strain CBDB1) GN=uvrB PE=3 SV=1 | bps03420 Nucleotide excision repair |
| Q63SA4 | UvrABC system protein C OS=Burkholderia pseudomallei (strain K96243) GN=uvrC PE=3 SV=2 | cbd03420 Nucleotide excision repair |
| A9KFP2 | UvrABC system protein C OS=Coxiella burnetii (strain Dugway 5J108-111) GN=uvrC PE=3 SV=1 | mms03420  Nucleotide excision repair |
| A6SXR9 | UvrABC system protein C OS=Janthinobacterium sp. (strain Marseille) GN=uvrC PE=3 SV=1 | bpy03420 Nucleotide excision repair |
| B2SZU5 | UvrABC system protein C OS=Paraburkholderia phytofirmans (strain DSM 17436 / LMG 22146 / PsJN) GN=uvrC PE=3 SV=1 | pol03420  Nucleotide excision repair |
| Q128Q7 | UvrABC system protein C OS=Polaromonas sp. (strain JS666 / ATCC BAA-500) GN=uvrC PE=3 SV=1 | vei03420 Nucleotide excision repair |
| A1WLD5 | UvrABC system protein C OS=Verminephrobacter eiseniae (strain EF01-2) GN=uvrC PE=3 SV=1 | afl00970  Aminoacyl-tRNA biosynthesis |
| B7GH39 | Valine--tRNA ligase OS=Anoxybacillus flavithermus (strain DSM 21510 / WK1) GN=valS PE=3 SV=1 | bcl00970  Aminoacyl-tRNA biosynthesis |
| Q5WEQ4 | Valine--tRNA ligase OS=Bacillus clausii (strain KSM-K16) GN=valS PE=3 SV=1 | bha00970  Aminoacyl-tRNA biosynthesis |
| Q9K8G8 | Valine--tRNA ligase OS=Bacillus halodurans (strain ATCC BAA-125 / DSM 18197 / FERM 7344 / JCM 9153 / C-125) GN=valS PE=3 SV=1 | bha00970  Aminoacyl-tRNA biosynthesis |
| Q65GK8 | Valine--tRNA ligase OS=Bacillus licheniformis (strain ATCC 14580 / DSM 13 / JCM 2505 / NBRC 12200 / NCIMB 9375 / NRRL NRS-1264 / Gibson 46) GN=valS PE=3 SV=1 | bld00970  Aminoacyl-tRNA biosynthesis |
| P11931 | Valine--tRNA ligase OS=Geobacillus stearothermophilus GN=valS PE=1 SV=1 | valyl-tRNA aminoacylation |
| A6SUQ8 | Valine--tRNA ligase OS=Janthinobacterium sp. (strain Marseille) GN=valS PE=3 SV=1 | mms00970  Aminoacyl-tRNA biosynthesis |
| Q8EPN2 | Valine--tRNA ligase OS=Oceanobacillus iheyensis (strain DSM 14371 / CIP 107618 / JCM 11309 / KCTC 3954 / HTE831) GN=valS PE=3 SV=1 | oih00970  Aminoacyl-tRNA biosynthesis |
| Q5N3J4 | Valine--tRNA ligase OS=Synechococcus sp. (strain ATCC 27144 / PCC 6301 / SAUG 1402/1) GN=valS PE=3 SV=1 | syc00970 Aminoacyl-tRNA biosynthesis |
| Q8DQN5 | Zinc metalloprotease ZmpB OS=Streptococcus pneumoniae (strain ATCC BAA-255 / R6) GN=zmpB PE=3 SV=1 | metabolism |
| Q9L7Q2 | Zinc metalloprotease ZmpB OS=Streptococcus pneumoniae serotype 4 (strain ATCC BAA-334 / TIGR4) GN=zmpB PE=3 SV=2 | metabolism |
